# The human Na^+^/H^+^ exchanger 1 is a membrane scaffold protein for extracellular signal-regulated kinase 2

**DOI:** 10.1186/s12915-016-0252-7

**Published:** 2016-04-15

**Authors:** Ruth Hendus-Altenburger, Elena Pedraz-Cuesta, Christina W. Olesen, Elena Papaleo, Jeff A. Schnell, Jonathan T. S. Hopper, Carol V. Robinson, Stine F. Pedersen, Birthe B. Kragelund

**Affiliations:** Cell and Developmental Biology, Department of Biology, University of Copenhagen, Universitetsparken 13, DK-2100 Copenhagen Ø, Denmark; Structural Biology and NMR Laboratory, Department of Biology, University of Copenhagen, Ole Maaløes Vej 5, DK-2200 Copenhagen N, Denmark; Physical and Theoretical Chemistry Laboratory, Department of Chemistry, University of Oxford, South Parks Road, Oxford, OX1 3QZ UK

**Keywords:** NHE1, Intrinsically disordered protein, Phosphorylation, MAPK, Shuffle complex, NMR, Scaffold

## Abstract

**Background:**

Extracellular signal-regulated kinase 2 (ERK2) is an S/T kinase with more than 200 known substrates, and with critical roles in regulation of cell growth and differentiation and currently no membrane proteins have been linked to ERK2 scaffolding.

**Methods and results:**

Here, we identify the human Na^+^/H^+^ exchanger 1 (hNHE1) as a membrane scaffold protein for ERK2 and show direct hNHE1-ERK1/2 interaction in cellular contexts. Using nuclear magnetic resonance (NMR) spectroscopy and immunofluorescence analysis we demonstrate that ERK2 scaffolding by hNHE1 occurs by one of three D-domains and by two non-canonical F-sites located in the disordered intracellular tail of hNHE1, mutation of which reduced cellular hNHE1-ERK1/2 co-localization, as well as reduced cellular ERK1/2 activation. Time-resolved NMR spectroscopy revealed that ERK2 phosphorylated the disordered tail of hNHE1 at six sites in vitro, in a distinct temporal order, with the phosphorylation rates at the individual sites being modulated by the docking sites in a distant dependent manner.

**Conclusions:**

This work characterizes a new type of scaffolding complex, which we term a “shuffle complex”, between the disordered hNHE1-tail and ERK2, and provides a molecular mechanism for the important ERK2 scaffolding function of the membrane protein hNHE1, which regulates the phosphorylation of both hNHE1 and ERK2.

**Electronic supplementary material:**

The online version of this article (doi:10.1186/s12915-016-0252-7) contains supplementary material, which is available to authorized users.

## Background

Extracellular signal-regulated kinase 2 (ERK2) is a member of the mitogen-activated protein kinase (MAPK) family of kinases activated in response to numerous growth factors and cytokines, leading to phosphorylation and functional regulation of downstream targets. ERK2 has been linked to more than 200 different substrates whose phosphorylation by ERK2 is orchestrated by coordination of signaling networks through common binding to so-called scaffold proteins [1]. The definition of a scaffold protein was recently refined and their identification as such suggested from qualities of multivalent binding, non-catalytic placeholders, and bidirectional process control [2]. Several scaffold proteins have been described for the MAPKs such as kinase suppressor of Ras (KSR) [3], JNK-interacting protein (JIP) [4], IQ motif containing GTPase activating protein 1 (IQGAP1) [5], and β-arrestin [6], which interact with members of the MAPK cascade, providing multivalency, spatial concentration, and/or signaling fidelity. However, although MAPKs are known to regulate the action of several membrane proteins and receptors, none of these scaffold proteins are themselves membrane proteins, requiring additional mechanisms for colocalization of the scaffold protein, the membrane protein, as well as the kinases. Moreover, most of the available molecular insights are from structures of kinases in complex with folded domains or with small peptides of the scaffold proteins, and details regarding scaffolding by non-globular proteins are lacking.

MAPKs are S/T kinases that interact with targets and regulators via two types of domains, D-domains and F-sites [7–11]. D-domains, also known as docking sites for ERK and JNK, LXL (DEJL) domains, or kinase interaction motifs (KIMs) have the canonical sequence of 2–5 basic residues (R/K), spaced by 1–6 residues to a hydrophobic motif ΦXΦ, where Φ is generally V, L, or I [8, 9]. D-domains are found in MAPK substrates such as the transcription factor Elk-1 and p90 ribosomal S kinase (RSK1-3), as well as in other MAPK targets [8, 9, 11]. In ERK2, D-domains interact with the D-domain recognition site also known as the CD/ED (common docking domain/glutamate/aspartate docking) domain, located more than 10 Å from the active site [8, 9, 11]. The F-site recruitment site in ERK2 is much less studied and incompletely understood. It binds to F-sites, also called DEF (docking site for ERK, FXFP)-domains with the canonical FXFP sequence [12]. F-sites allow for aromatic residues at the P1 (F, W) and P3 positions (F, Y, W) [13], and F-sites have been reported in substrates such as Elk-1 (FQFP) [14] and c-Fos (FTYP) [15], and within the nucleoporin FG-repeats (FXFG) [16, 17]. So far the only structure available of an F-site recruitment site-interacting protein is that of ERK2 in complex with the 15 kDa phosphoprotein enriched in astrocytes (PEA-15), which notably lacks any of the above-mentioned motifs [18].

The plasma membrane Na^+^/H^+^ exchanger 1 (NHE1, SLC9A1) is a major regulator of pH and volume in essentially all cells studied. Furthermore, NHE1 is involved in the regulation of cell proliferation, survival, motility, and other essential physiological processes, and its dysregulation contributes importantly to major human malignancies, including cancer and cardiovascular diseases [19, 20]. Numerous hormones and growth factors acting via receptor tyrosine kinases or GTP-binding protein-coupled receptors can elicit posttranslational regulation of NHE1 [21–23]. The MAPKs ERK1/2, p38 MAPK, and c-Jun N-terminal kinase (JNK) are widely implicated in NHE1 regulation [24–28], and direct phosphorylation of human (h) NHE1 by ERK1/2 was previously proposed based on ^32^P measurements [25] and mass spectrometry [29]. Conversely, NHE1 has been reported to regulate signaling through regulation of ERK1/2 and p38 MAPK activity [26, 28, 30–32], and yeast two-hybrid screens have suggested the interaction of NHE1 with several members of the MAPK hierarchy [33]. However, with the exception of the interaction with B-Raf [34], evidence from mammalian systems is lacking, and the possible sites of NHE1-MAPK interaction, its structural details, and possible functional consequences are unexplored. We recently showed by PONDR and DISOPRED predictions, as well as by nuclear magnetic resonance (NMR) spectroscopy and other biophysical techniques, that the distal ~ 130 residues of the hNHE1 C-terminal intracellular domain (hNHE1cdt), containing most of the known NHE1 phosphorylation sites, are intrinsically disordered (ID) [35, 36]. To our knowledge, no studies have yet addressed the mechanisms through which MAPKs interact with ID proteins (IDPs), although about one third of all proteins in higher eukaryotes contain significant ID regions (IDRs) [37], and ID is abundant in cellular signalling [38], scaffolding [39], as well as in MAPKs themselves [40].

Here, we demonstrate that hNHE1 acts as an ERK2 membrane protein scaffold *in vivo* that is necessary for ERK2 activation via direct interactions, and we show that loss of scaffolding by hNHE1 leads to decreased ERK2 activation. Using NMR spectroscopy we show that NHE1 scaffolds inactive (ia) ERK2 in a “shuffle complex” that involves a D-domain and two non-canonical F-sites. We characterize the order and kinetics of both previously reported and novel ERK2-mediated phosphorylations of hNHE1 *in vitro*. Our findings provide a molecular mechanism for the widely recognized and functionally important scaffolding function of hNHE1, and give mechanistic insight into the regulation of ERK2 activity by the intrinsically disordered hNHE1cdt.

## Results

**Fig. 1:**
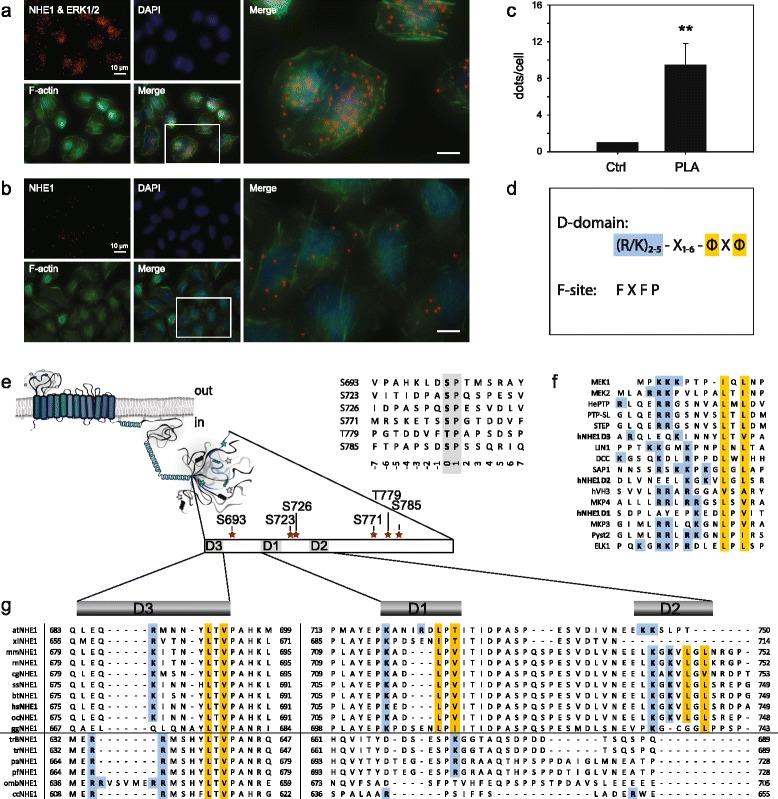
Direct interaction between ERK2 and NHE1 *in vivo* and *in silico.* **a** Proximity ligase assay carried out in AP-1 WT hNHE1 cells treated with NHE1 and ERK1/2 primary antibodies. Proximity ligase signal appears as *red dots* and the *merge image* highlights the interaction between NHE1 and ERK. F-actin was stained with phalloidin^488^. Scale bars represent 10 μm. Data are representative of three independent replicates. **b** Proximity ligase assay carried out in AP-1 WT hNHE1 cells. As a negative control, cells were only treated with NHE1 primary antibodies and the appearance of the *red dots* indicates unspecific binding. F-actin was stained with phalloidin^488^. Scale bars represent 10 μm. Data are representative of three independent replicates. **c** Quantification of PLA data was carried out in ImageJ. PLA signal from at least ten different image areas in each experiment were counted by particle analysis and the average PLA signal per cell was plotted in the bar graph. Data are representative of three independent replicates. **d** ERK1/2 docking motifs, D-domain and F-site. *Φ* indicates hydrophobic amino acid residues (typically L, V, or I), and *X* any other amino acid. **e** Overall NHE1 topology and localization of putative D-domains and ERK2 phosphorylation sites in the hNHE1cdt. The positions of identified D-domains as well as predicted (S/T)P-phosphorylation sites are indicated by *stars. Insert*: alignment of the consensus ERK2 phosphorylation sites in hNHE1; (S/T)P sites indicated by *grey background*. **f** Alignment of hNHE1 D-domains to known D-domains. The consensus hydrophobic and positively charged residues are highlighted in *yellow* and *blue*, respectively. **g** Sequence conservation of putative D-domains in NHE1cdt in various species. D1, D2, and D3 are indicated with *grey bars* above the alignment. The *solid horizontal line* separates tetrapods (*top*) from teleosts (*bottom*) [35]

The recently suggested links between hNHE1 and ERK1/2 prompted us to investigate whether ERK1/2 and NHE1 directly interact in a cellular context. Using AP-1 cells (which lack endogenous NHE1 [41]) stably expressing full-length WT hNHE1, we asked if hNHE1 and ERK1/2 engage in a direct interaction *in vivo.* Interaction was assessed using *in situ* proximity ligation assay (PLA), which interrogates close interaction (<40 nm) between proteins (Fig. 1). As seen, the detection of multiple PLA puncta when cells were incubated with both NHE1 and ERK1/2 antibodies revealed the presence of hNHE1-ERK1/2 complexes in AP-1 WT hNHE1 cells (Fig. 1a), compared to a much lower signal in negative controls incubated with one antibody only (Fig. 1b). Data from multiple experiments are quantified in Fig. 1c, demonstrating that the PLA signal is significantly greater in NHE1-ERK antibody-labelled cells than in negative controls. Thus, these data show that hNHE1 and ERK1/2 directly interact in the cell.

### The disordered tail of hNHE1 interacts with iaERK2

**Fig. 2:**
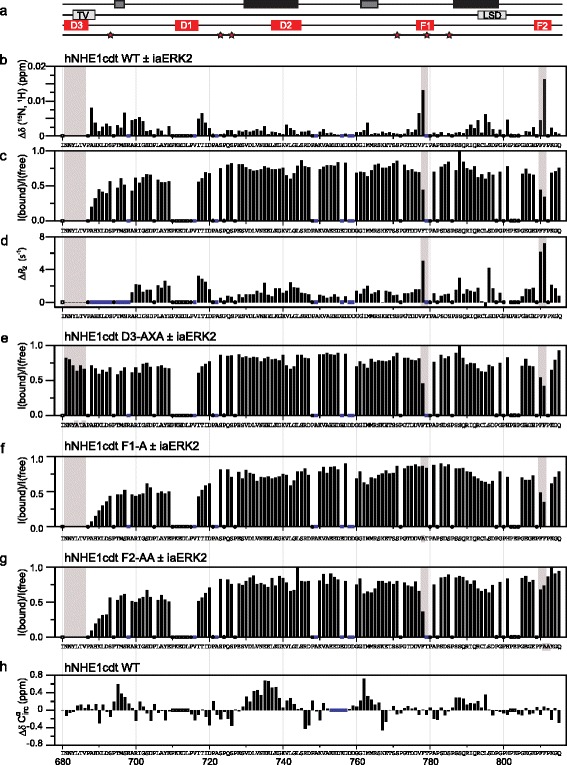
Inactive ERK2 interacts preferably with the D3-domain and two F-sites of hNHE1cdt. **a** Position of transient helices (*black* and *dark grey bars*, see panel **h**), TV- and LSD-boxes (*light grey bars*), predicted D-domains and F-sites (*red bars*), as well as ERK2 phosphorylation sites (*stars*) in the hNHE1cdt*.* **b** Combined chemical shift perturbations Δδ(^15^N,^1^H) of WT hNHE1cdt by iaERK2 interaction. **c** ^15^N,^1^H-HSQC peak intensity ratios of hNHE1cdt WT in the presence/absence of iaERK2. **d** Difference in relaxation rates Δ*R*_*2*_ between hNHE1cdt and NHE1cdt:iaERK2. **e–g** ^15^N,^1^H-HSQC peak intensity ratios of **e** hNHE1cdt D3-AXA, **f** hNHE1cdt F1-A, and **g** hNHE1cdt F2-AA in the presence/absence of iaERK2. **h** Internally urea referenced secondary C^α^ chemical shifts (ΔδC^α^) of WT hNHE1cdt identify the presence of several transient helices (ΔδC’ in Additional file 2: Figure S2h). *●* indicate the position of prolines, *□* unassigned residues, and *blue box* severe peak overlap

Scrutinizing the intracellular domain of NHE1 by *in silico* methods for potential ERK1/2 interaction sites identified three potential D-domains in the intrinsically disordered region (IDR) [35, 36], [LAYEPKEDLPVITIDP]_706–721_ (D1), [LVNEELKGKVLGLSR]_732–746_ (D2), and [LEQKINNYLTVPA]_676–688_ (D3) (listed in the order of stringency) (Fig. 1d–g). The previously described conserved TV-box is part of the D3-domain [35], and this D-domain is the only one conserved throughout NHE1 evolution (Fig. 1g). Since ERK1 and ERK2 are 84 % identical by sequence and share many if not all functions [42], and ERK2 is the more widely studied enzyme of the two, we focused *in vitro* studies on ERK2. To discriminate between the roles of each D-domain, we investigated the interaction between the disordered tail of NHE1 (residues I680-Q815 (hNHE1cdt)) and recombinantly produced human iaERK2 by NMR spectroscopy, which previously had provided insight into the transient structure and conserved regions of hNHE1cdt [35] (Fig. 2a). First, we measured perturbations of chemical shifts and peak intensities of the hNHE1cdt WT arising from addition of iaERK2 to a 1:1 molar ratio using ^15^N,^1^H-HSQC spectra (Fig. 2b, c). Signals from all residues of the D3-domain disappeared, and decreased intensities and chemical shift perturbations were observed in the C-terminal neighbouring residues, suggesting this domain engages in the interaction. Additionally, two Phe residues in the distal end of hNHE1cdt [PFFPKGQ]_809–815_, as well as a Phe residue within a potential substrate site, [FTP]_778–780_, were highly perturbed. Importantly, although the latter resembles a substrate site, both are reminiscent of the canonical ERK F-site motif, suggesting several interaction sites between hNHE1cdt and iaERK2. Additional yet minor perturbations were observed in the linking regions between these sites, which were partially caused by slight pH variations (Additional file 1: Figure S1a). Since some residues in D1 are unassigned due to their overlap in the NMR spectra, and perturbations were observed close to this site (Fig. 2b, c), we cannot exclude D1 to also contribute to the interaction. Lastly, as *Escherichia coli* expression of ERK2 can lead to autophosphorylation of Y187 [43], we assessed the level of ERK2 autophosphorylation by native PAGE, showing that more than 80 % of ERK2 is non- and less than 20 % mono-phosphorylated (Additional file 1: Figure S1b). To ensure that this had no effect on the interaction with NHE1cdt we fully dephosphorylated ERK2 with the Tyr phosphatase HePTP and re-analysed the interaction, which gave an identical binding profile (Additional file 2: Figure S2a, b), in accordance with the low activity of the ERK2 mono-phosphorylated state [44]. In conclusion, NHE1cdt interact with iaERK2 through multiple contact sites involving the D3-domain, as the dominating D-domain, and two F-sites (F1, FTP_780_ and F2, FFP_811_).

We next investigated whether interfering with any of these contact sites would affect the interactions and first exploited the knowledge that MAPK interaction is severely perturbed by mutations of ΦXΦ to AXA in D-domains [45]. Hence, we constructed AXA variants of all three D-domains alone and in combination, both in the full-length hNHE1 (hNHE1-D1-AXA, hNHE1-D2-AXA, etc.) for cellular studies and in hNHE1cdt (D1-AXA, D2-AXA, D3-AXA) for *in vitro* studies. In D3-AXA, chemical shift perturbations in the two F-sites upon ERK2 addition were preserved in the interaction with iaERK2, whereas no perturbations were observed in the AXA-mutated D3-domain, implying that this site is important for the interaction (Fig. 2e and Additional file 2: Figure S2c). No notable effects of D1- and D2-AXA substitutions were observed (Additional file 2: Figure S2d–e), arguing against their involvement in the interactions. This data also indicated that the F-sites interacted with iaERK2 independently of the D-domain. To assess this further the F-sites were individually mutated by substituting FTP_778–780_ with ATP_778–780_ (denoting the F1-A variant) and FP_811–812_ with AA_811–812_ (denoting the F2-AA variant). Both F-site variants showed strongly decreased chemical shift perturbations at the mutation sites upon ERK2 addition, leaving the other F-site and the D3-domain unaffected (Fig. 2f–g and Additional file 2: Figure S2f–g). This conclusively identified all three sites as ERK2 interaction sites. Further, substitutions at each site left the other sites unaffected, indicating that these regions of hNHE1 interact independently with iaERK2.

### NHE1 does not fold upon binding to ERK2 but may be a flexible wrapper

To further address how hNHE1cdt interacted with iaERK2 we analysed the complex by size exclusion chromatography (SEC) and compared elution profiles with those of the individual proteins (Additional file 3: Figure S3a–c). Since hNHE1cdt is an IDR, it has a larger hydrodynamic radius than iaERK, and hNHE1cdt thus eluted first from the column. Subtracting individual runs from that of the mixture revealed a broad peak with an elution volume smaller than that of iaERK2, yet larger than that of hNHE1cdt, suggesting that hNHE1cdt folds or wraps around iaERK2. Circular dichroism (CD) spectroscopic analyses (Additional file 3: Figure S3d), as well as NMR chemical shift analyses (Fig. 2b), did not indicate folding upon binding formation of significant secondary structure, suggesting that hNHE1cdt forms a relatively extended structure around iaERK2. To substantiate this conclusion, we recorded ^15^N transverse relaxation rates of the unbound (*R*_2_^free^) and the iaERK2-bound hNHE1cdt (*R*_2_^bound^), and analysed their differences (Fig. 2d). Since D3 residues broadened beyond detection in the complex, their *R*_*2*_ values could not be extracted. However, for residues interacting with iaERK2, a significant increase in *R*_2_ rates is expected compared to those of hNHE1cdt alone, due to the larger radius of gyration of the complex or due to chemical exchange between different states. Indeed, residues from both F-sites had substantially larger *R*_2_ rates in the complex compared to hNHE1cdt alone, and many residues between these sites were also affected, although not to the same extent. This result supports the SEC results and suggests a substantial interaction area between the two proteins.

**Fig. 3:**
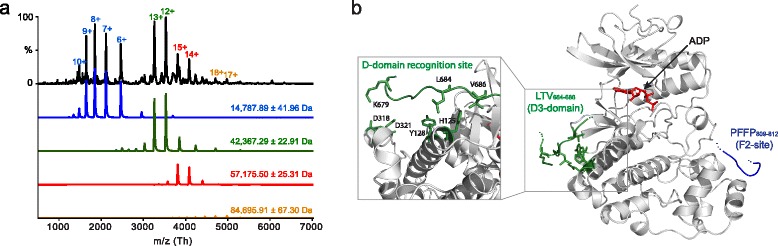
Native mass spectrum (MS) and molecular model of the hNHE1cdt:iaERK2 complex. **a** Non-denaturing mass spectrum of the hNHE1-ERK2 complex, acquired from 200 mM ammonium acetate. Deconvolution was performed using the UniDec software and the individual charge series fits are displayed *below*, corresponding to NHE1 (*blue*), ERK2 (*green*), NHE1:ERK2 complex (*red*), and minor contribution of ERK2 dimer (*orange*). **b** Proposed model for the hNHE1-ERK2 interactions. The hNHE1cdt interacts with ERK2 at three sites, exploiting a D-domain and two F-sites (shown in *green* and *blue*, respectively). At the D-domain recognition site, K679, L684, and V686 of the hNHE1cdt D3-domain, as well as D318, D321, Y128, and H125 of the iaERK2 D-domain recognition site are shown as *sticks*

The loss of NMR signal upon binding imposes a challenge for obtaining affinity constants. Although NMR peak intensities are only directly proportional to the populations when all states can be accounted for, they may provide apparent upper-limit affinities. From a titration of hNHE1cdt with iaERK2, global fitting of intensity changes using residues LTV_684–686_ of the D3-domain gave a K_d_^app^ of 16 ± 2 μM, while the other interaction sites had relatively lower affinities with apparent affinities, K_d_^app^ of 86 ± 26 μM (F1, F778) and 69 ± 14 μM (F2, F811) (Additional file 4: Figure S4). Attempts to substantiate this and obtain stoichiometries by isothermal titration calorimetry or microscale thermophoresis were unsuccessful, due to limited stability of concentrated ERK2, as well as adhesion of ERK2 to the capillary tubing. Instead, to determine the stoichiometry of the interaction we used non-denaturing mass spectrometry, allowing the non-covalent complex to be captured at low micromolar concentration. Deconvolution of the spectrum using in-house software [46] revealed three main charge state envelopes, corresponding to hNHE1cdt (14,787.89 Da), iaERK2 (42,367.39 Da), and from the 1:1 complex (57,175.50 Da) (Fig. 3a). A minor population of iaERK2 dimers was also detected (84,695.91 Da), most likely due to the presence of a hexahistidine-tag, as noted [47]. These data unequivocally showed that hNHE1cdt and iaERK2 form a 1:1 complex, fully in-line with the perturbation of the relaxation times and the size exclusion data. Collectively, these data indicated that NHE1cdt binds to ERK2 with medium affinity, through at least three independent contact sites forming a 1:1 complex.

Finally, we used the NMR chemical shifts and relaxation data together with known structures of iaERK2 complexes to model the hNHE1cdt-iaERK2 interaction (Fig. 3b). When modelled at the D-domain recognition site of iaERK2, each individual D-domain (D1, D2, or D3) when bound to the D-domain recognition site allowed for either of the F-sites (F1 or F2) to reach the F-site recognition site. In each case the model predicted long ID linkers between the binding sites, which were long enough to allow NHE1 to wrap around iaERK2. This is in accordance with the NMR data and supports their uncoupled behaviour. Collectively, these data suggest that hNHE1cdt could interact with iaERK2 in a tripartite 1:1 interaction exploiting a D-domain (D3) and two F-sites.

### ERK2 phosphorylates hNHE1cdt at six consensus sites in a distinct order and with different kinetics

**Fig. 4:**
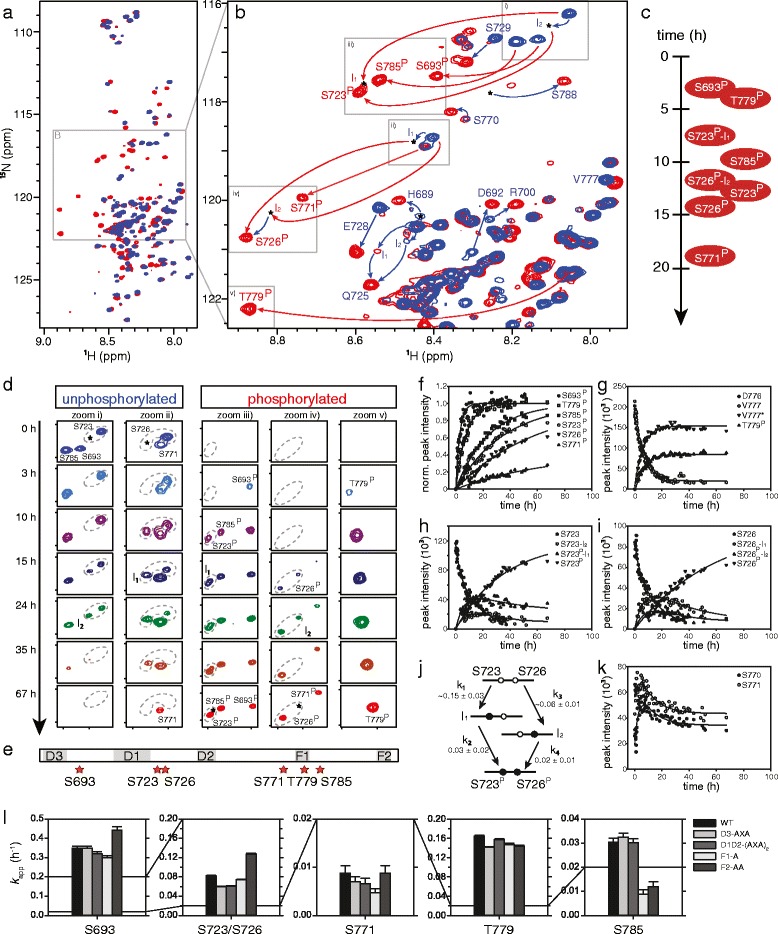
Active ERK2 phosphorylates six (S/T)P-sites in hNHE1cdt in a distinct order and with different kinetics. **a** ^15^N,^1^H-HSQC spectra of unphosphorylated (*blue*) and ERK2 phosphorylated hNHE1cdt WT (*red*). Addition of catalytic amounts of aERK2 to ^15^N-labelled hNHE1cdt resulted in diagnostic changes in chemical shifts evident of specific S/T-phosphorylation events. **b** Zoom on S/T and S^P^/T^P^ regions of **a**. *Red labels/arrows* indicate phosphorylation-induced peak shifts, and *blue arrows/labels* peak shifts of neighbouring residues sensing phosphorylation. **c** Time courses of hNHE1cdt phosphorylations and order of peak appearances. **d** Phosphorylation time courses. Zoom i–v on boxes in **b**. Peaks of unphosphorylated (*left panel*) and phosphorylated states (*right panel*) simultaneously disappear/appear with time, in a distinct order. *Dashed lines* encircle multiple peaks of the same residue reporting on distinct phosphorylation states. *Stars* indicate position of intermediates. **e** Relative positions of ERK2 phosphorylation sites (*stars*) to D-domains and the F-sites in hNHE1cdt **f** Changes of peak intensities with time reported on the phosphorylation rates at the individual sites. S693 and T779 were the first and most reactive aERK2 phosphorylation sites in hNHE1cdt, followed by S723 (I_1_), then S785 simultaneously with S726 (I_2_), followed by dually phosphorylated S723 and S726, and lastly S771. **g** Kinetics of T779 phosphorylation shown by peak disappearance of D776 and V777 concomitant with peak appearance for V777* and T779^P^. Apparent rate constants can be extracted by fitting the unphosphorylated disappearing peak or the appearing phosphorylated peak of either the phosphorylated residue or close neighbours, and should, for a two-state reaction, be the same irrespective of which peak is used for fitting. Two-state behaviour was observed for S693 and T779. **h–j** Phosphorylation of S723 and S726 showed more complex behaviour with interlinked rates. These residues are so close that a neighbouring phosphorylation event would influence their chemical shifts, leading to the observation of intermediates, i.e. phosphorylation of the neighbour in the self-unphosphorylated state, and vice versa (see also panel **d**). The observation of two intermediates I_1_ and I_2_ suggested parallel phosphorylation of S723 and S726. Both peaks of the dually unphosphorylated state disappeared with faster rates than the final peaks from the dually phosphorylated state appeared. Thus, the apparent phosphorylation rates of each site were highly dependent on the phosphorylation state of the neighbour, i.e. *k*_1_ and *k*_4_ as well as *k*_2_ and *k*_3_ are not identical. I_1_ appeared first with a rate similar to the fast decay of the unphosphorylated states (*k*_1_, see also Table 1), whereas I_2_ appeared concomitantly with the dually phosphorylated state but much slower (*k*_2_ and *k*_3_) and with low intensities. Thus, although both orders of phosphorylation were observed, the main path was via intermediate I_1_, i.e. phosphorylation of S723 first, followed by phosphorylation of S726. **k** The weak peaks reporting on the unphosphorylated states of S771 and S785 initially gained intensity before starting to decrease due to phosphorylation (shown for S771). As peak intensities are strongly dependent on dynamics, this observed increase may result from altered dynamics caused by the nearby phosphorylation of T779. **l** Apparent rate constants for the individual phosphorylation sites and the effect of D-domain and F-site mutations. Docking site mutations do not change the order of phosphorylation events, yet modulate the individual rates in a distant dependent manner. Based on single measurements and standard deviations from the exponential fits, the apparent rates are significantly different except for S693 WT compared to D3-AXA, S771 WT compared to F2-AA, and S785 WT compared to D1D2-(AXA)_2_ (one-way ANOVA)

*In silico* analysis predicted six canonical ERK2 substrate sites in hNHE1cdt (Fig. 1e), several of which were previously shown to be phosphorylated *in vivo* [48] and *in vitro* [29]. We therefore used active ERK2 (aERK2) and time-resolved (TR) NMR spectroscopy to investigate whether hNHE1cdt could be phosphorylated by aERK2 *in vitro*, and how the individual D-domains and F-sites might contribute to this. Addition of catalytic amounts of aERK2 to ^15^N-labelled hNHE1cdt resulted in changes in chemical shifts diagnostic of specific S/T-phosphorylation events [49, 50] (Fig. 4a, b). From NMR assignments of the fully phosphorylated state of hNHE1, we identified all six sites (S693, S723, S726, S771, T779, and S785) to be phosphorylated in a distinct temporal order and with different rate constants (Fig. 4c–k). Phosphorylated (^P^) residues S693^P^ and T779^P^ appeared first and simultaneously. After a lag phase, when S693^P^ and T779^P^ had essentially reached saturation, S785^P^, S723^P^, S726^P^, and S771^P^ appeared in close succession (Fig. 4d, f). S771^P^ was the last to appear and did not reach saturation in the time frame of the experiment. Two-state behaviour was observed for S693 and T779 (shown for T779 in Fig. 4g), whereas phosphorylation of S723 and S726 showed more complex behaviour with interlinked rates. This can be inferred from the observation of two intermediates, i.e. phosphorylation of the neighbour in the self-unphosphorylated state, and *vice versa* (Fig. 4d, h–j). To determine if S693 and T779 acted as priming sites for subsequent phosphorylations of the four other sites, these were mutated to alanines in two different variants (S693A and T779A) and the phosphorylation kinetics repeated. The same order of phosphorylation was observed (Additional file 5: Figure S5a, b), which indicated that the relative order of phosphorylation depended solely on substrate specificity and/or recognition domain affinity, and that neither S693 or T779 acted as priming sites. In summary, these results demonstrate complex behaviour of hNHE1cdt phosphorylation by aERK2, with a defined order, and additional intermediate states. S693 and T779 were the first and most reactive aERK2 phosphorylation sites in hNHE1cdt, but did not act as priming sites, followed by S723 (I_1_), then S785 simultaneously with S726 (I_2_), followed by dually phosphorylated S723 and S726, and lastly S771.

### D-domains play differential roles in scaffolding and activation

We subsequently asked whether the D-domains and F-sites affected the NHE1 phosphorylation pattern *in vitro*, as well as the scaffolding function *in vivo*. All sites were analysed *in vitro* using the AXA mutations of the D-domains (D3 alone and D1D2 combined) and alanine mutations of the F-sites, and were analysed together with WT hNHE1 using the same batch of aERK. For all variants, all six S/T-sites were phosphorylated in the same order as in the WT hNHE1, but with altered kinetics (Fig. 4l, Table 1, and Additional file 5: Figure S5c). Analysing those data is not trivial. Since S723 and S726, as well as S771, T779, and S785 are close in sequence, they sense the phosphorylation state of the neighbour, leading to intermediate states with altered dynamics and/or chemical environment. The T779 phosphorylation site overlaps with the F1-site, so the effect of F1 mutation may be either due to the loss of the interaction or due to the altered intrinsic affinity of the phosphosite. Also, the slow phosphorylation sites S771 and S785 may be affected by altered kinetics of the fast sites due to simple intramolecular substrate competition.

**Table 1:** Apparent rate constants, *k*_app_ for hNHE1cdt phosphorylation by aERK2, and effect of D-domain and F-site mutations

P-site	*k*_app_ (h^-1^)				
	WT	D3-AXA	D1D2-(AXA)_2_	F1-A	F2-AA
S693	0.3489 ± 0.0101	0.3488 ± 0.0112	0.3193 ± 0.0109	0.3000 ± 0.009	0.4435 ± 0.0177
S723/S726^a^	0.0821 ± 0.0018	0.0592 ± 0.0010	0.0610 ± 0.0011	0.0741 ± 0.0015	0.1274 ± 0.0018
S771	0.0088 ± 0.0015	0.0070 ± 0.0011	0.0066 ± 0.0011	0.0047 ± 0.0008	0.0088 ± 0.0015
T779	0.1648 ± 0.0022	0.1413 ± 0.0017	0.1572 ± 0.0021	0.1473 ± 0.0024	0.1441 ± 0.0018
S785	0.0305 ± 0.0016	0.0325 ± 0.0016	0.0302 ± 0.0017	0.0088 ± 0.0019	0.0120 ± 0.0021

^a^Fitting of S723 and S726 individually would require fitting to biexponentials due to their extensive crosstalk, which in turn reduces fitting accuracy. Therefore, only decrease of the S723 peak intensity was fitted, which reports on both S723 and S726 phosphorylation due to their crosstalk

In detail, S693 phosphorylation was not affected by the D3-AXA mutation, but was slowed down by the D1D2-(AXA)_2_ and F1-A variants, and accelerated by the most distant F2-AA variant. Similarly, S723/S726 phosphorylations were slowed down by all mutations except the most distant one, F2-AA that again led to accelerated rates. T779 phosphorylation was slowed down by all mutations, and S785 was strongly decreased by both F-site mutants. Taken together, mutations of the D-domains and F-sites affected phosphorylation in a distance dependent manner. In three cases (S693 and S723/S726 phosphorylation of the F2-AA variant) the rate of phosphorylation went up, suggesting that the presence of this site was inhibitory. In all three cases this occurred for the site furthest away from the mutation, reflecting competition between sites. This suggests that the D-domains and F-sites are not mandatory for phosphorylation, but rather exert regulatory roles, and that each site uses the most optimal ERK2 interaction site to become phosphorylated.

**Fig. 5:**
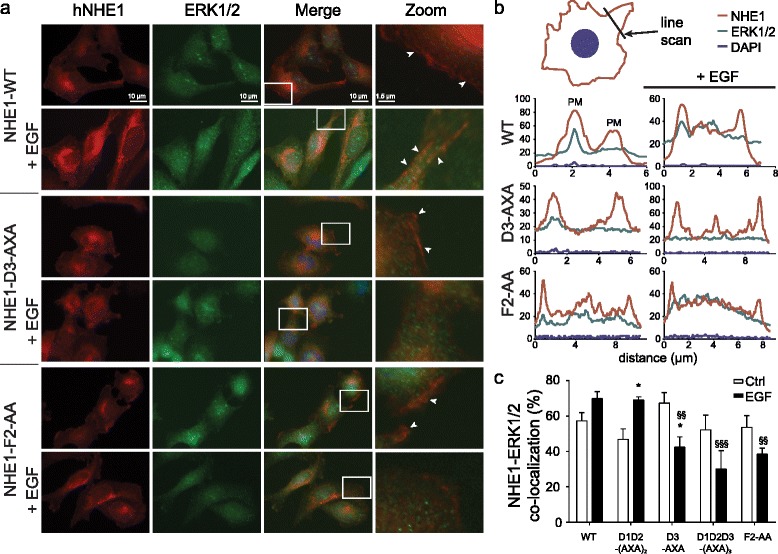
NHE1 co-localizes with ERK2 shown by immunofluorescence. **a** Immunofluorescence images of AP-1 cells (hNHE1 WT, D3-AXA and F2-AA) treated or not for 15 min with EGF (100 ng/ml). *Merged images* were zoomed to highlight the co-localization of ERK1/2 and NHE1 (*white arrowheads*). All other variants are shown in Additional file 6: Figure S6. Data are representative of three independent biological replicates. **b** Representative line scans across membrane areas of images as in **a**. Line scans were performed using Olympus image analysis software. The figure shows the pixel intensities at each wavelength over the line shown, in the absence and presence of EGF as shown. Data are examples based on analysis of at least 60 cells in three to four independent replicates per condition. **c** Summary of line scan analysis data for all variants. The figure shows the percentage of cells with NHE1-ERK1/2 co-localization in both membranes, based on the experiments illustrated in **b**. Data are shown as mean percentage with SEM error bars, based on analysis of at least 60 cells in three to five independent replicates per condition. §§ and §§§, *p* < 0.01 and *p* < 0.001, respectively, relative to AP1 + EGF; * *p* < 0.05 relative to own control in absence of EGF. Two-way ANOVA, Šídák’s multiple comparisons test

To determine whether the D-domain and F-site variants affected hNHE1-ERK2 interaction in a cellular context we used immunofluorescence analysis, which revealed that hNHE1 localized predominantly to the plasma membrane region of AP-1 cells, although some intracellular hNHE1 labeling was also seen (Fig. 5a), fully consistent with previous reports [22, 51] and with the PLA studies (Fig. 1a, b). Stimulation with epidermal growth factor (EGF, 100 ng/ml) induced distinct plasma membrane ruffles, to which hNHE1 clearly localized, in congruence with its role in cell motility [52, 53], and similar to the localization of NHE1 to prolactin-induced ruffles, which we recently demonstrated [54]. ERK1/2 localized diffusely in the cytosol and nucleus, as well as in plasma membrane regions, where it partially co-localized with hNHE1 (white arrowheads). In a cellular context we refrained from using the F1-variant, as the phenylalanine is part of a phosphorylation site (FTP_778–780_), thus complicating the interpretations. Similar to WT hNHE1, all hNHE1 variants localized predominantly to the plasma membrane region, suggesting that the mutations did not affect NHE1 membrane targeting (Fig. 5 and Additional file 6: Figure S6). Compared to cells expressing WT hNHE1, co-localization with ERK1/2 was unaltered both under basal and stimulated conditions in cells expressing hNHE1-D1D2-(AXA)_2_ (Fig. 5c and Additional file 6: Figure S6b), suggesting that D1 and D2 are not important for *in vivo* scaffolding and in congruence with their role in phosphorylation (Fig. 4l). Interestingly, whereas stimulation by EGF tended to increase co-localization in cells expressing WT hNHE1 and D1D2-(AXA)_2_, the opposite was true in cells expressing the D3-AXA, D1D2D3-(AXA)_3_, and F2-AA-variants (Fig. 5b, c and Additional file 6: Figure S6a, b). Hence, in these variants, a loss of colocalization was seen, which suggests that scaffolding as well as the dynamics of the hNHE1-ERK2 interaction are dependent on both the D3-domain and the F2-site. These data fully support the *in vitro* NMR data and highlight both the D3-domain and F2-site as important for scaffolding.

### NHE1 regulates ERK2 phosphorylation status in a cellular context

**Fig. 6:**
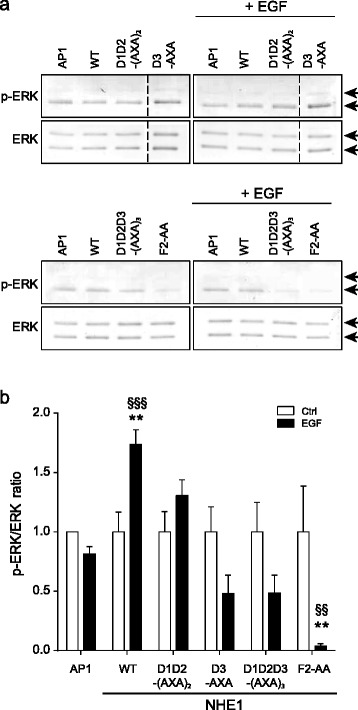
NHE1 regulates the phosphorylation status of cellular ERK1/2. ERK1/2 phosphorylation in untransfected AP-1 cells or AP-1 cells expressing WT and variant hNHE1, as indicated. Cells were stimulated or not with human recombinant EGF (100 ng/ml) for 15 min. **a** Representative immunoblots of p-ERK1/2 (T202/Y204 (ERK1)-T185/Y187 (ERK2) phosphorylation) and total ERK1/2 under the conditions shown. The *arrows* indicate ERK1 (*top*) and ERK2 (*bottom*), respectively. **b** Summary data from three to eight independent biological replicates per condition. Blots were scanned, and band intensities quantified using Un-Scan-IT Graph Digitizer software (Silk Scientific), as described in the “Methods”. Quantified data are shown as means with SEM error bars, normalized to those in untransfected, unstimulated AP-1 cells. *, **, and ***, *p* < 0.05, *p* < 0.01, and *p* < 0.001, respectively, compared to untransfected control; #, ##, and ###, *p* < 0.05, *p* < 0.01, and *p* < 0.001, respectively, compared to the same cells in absence of EGF. Two-way ANOVA, Holm-Šídák’s multiple comparisons test

To address whether NHE1 regulates ERK2 activity, we next assessed ERK1/2 activation by determining relative T202/Y204 (ERK1)-T185/Y187 (ERK2) phosphorylation, corresponding to ERK1/2 activation. Untransfected AP-1 cells, or AP-1 cells stably expressing WT hNHE1, were exposed to EGF (100 ng/ml) to induce ERK1/2 activity (e.g. [55]; Fig. 6a, b). As seen, despite the known low level of EGF receptor expression in Chinese hamster ovary (CHO)-derived cells such as AP-1 cells, stimulation of cells expressing WT hNHE1 with EGF evoked a modest but significant increase in ERK1/2 phosphorylation at these sites, i.e. ERK1/2 activation. Importantly, the presence of WT hNHE1 was necessary for detectable activation of ERK1/2 by EGF. This suggests that hNHE1 is important for ERK1/2 activation in this context. To assess whether the interaction-site mutations would affect NHE1-regulated ERK2 activity, we exposed untransfected AP-1 cells or AP-1 cells expressing WT and variant hNHE1s, to EGF and determined ERK1/2 activity ([55]; Fig. 6a, b). Compared to cells expressing WT hNHE1, cells expressing hNHE1-D3-AXA, hNHE1-D1D2D3-(AXA)_3_, or hNHE1-F2-AA variants exhibited a significant decrease in relative EGF-induced ERK1/2 phosphorylation (pERK1/2; Fig. 6b). These data show that hNHE1 is important for EGF-induced ERK1/2 activation in a cellular context, in a manner dependent on its D3-domain and F2-site. These data further support that this at least in part reflects a scaffolding role of hNHE1 involving the three independent low affinity sites. Notably, the effects of the mutations on ERK1/2-NHE1 co-localization and ERK1/2 activity are comparable if not identical, and mirrors the interaction monitored by NMR spectroscopy.

## Discussion

NHE1 activity after various stimuli is regulated by ERK1/2, and the NHE1 C-terminal tail is directly phosphorylated by ERK2 *in vitro* [29] and *in vivo* [25]. Vice versa, NHE1 can regulate ERK1/2 activity [26, 28, 30–32], yet molecular details and mechanistic understanding of their interaction have been lacking. In conjunction with yeast two-hybrid studies suggesting interaction of NHE1 with MAPKs [33], these studies led us to hypothesize that NHE1 and ERK2 engage in direct physical interaction. Supporting this hypothesis, we report here that ERK2 and NHE1 interact directly *in vivo* as well as *in vitro*, and that ERK2 phosphorylates multiple sites in hNHE1cdt. Based on NMR analyses, in conjunction with various combinations of D-domain and F-site mutations, we suggest that hNHE1cdt scaffolds iaERK2 and that they interact in a non-cooperative modular manner that involves a D-domain (D3) and two F-sites. NMR titrations revealed the affinity of the D3-domain to be in the low micromolar range, in agreement with known D-domain affinities [7]. D-domain and F-site mutations did not prevent ERK2-mediated hNHE1 phosphorylation *in vitro*, but altered its kinetics. *In vivo*, hNHE1 and ERK1/2 co-localized at the plasma membrane in a manner sensitive to ERK1/2 stimulation, and mutations in the hNHE1 D3-domain and F-site altered ERK1/2 activity. Thus, a central conclusion of this work is that NHE1 and ERK2 directly interact and engage in physical and functional reciprocal interactions. This provides a novel molecular framework for understanding previous reports of both NHE1-mediated scaffolding and regulation of ERK1/2 [28] and ERK1/2-mediated phosphorylation of NHE1 [25].

**Fig. 7:**
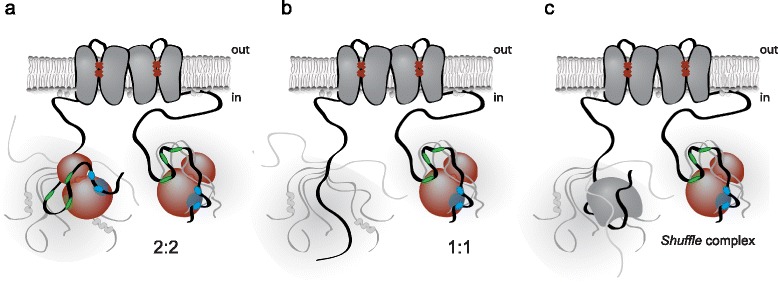
Scaffolding of ERK2 by the intrinsically disordered NHE1cdt via a “shuffle complex”. Suggested topology of the hNHE1cdt-iaERK2 complex indicating the tripartite binding mode between hNHE1cdt and human iaERK2. **a**–**b** As NHE1 functions as a dimer in the cell, one or both NHE1s in a dimer can shuffle an ERK1/2. **c** Alternatively, one NHE1 binds ERK1/2, whereas the other NHE1 in the dimer can scaffold other members of the signalling complex. Alternating conformations are indicated encompassing the individual sites and combinations

The organization of IDPs as “flexible wrappers” has previously been suggested as a general scaffolding mechanism [56], involved in bipartite interactions, for example of Ste5 with the yeast MAPK Fus3 [57] and of p21^CIP1^ with cyclin-dependent kinases [58]. Upon identification of the F-site, a bipartite modular, non-cooperative recognition system was originally suggested for kinases with the D-domain and F-site acting independently or in combination [12]. The tripartite (D-domain and two F-sites) interaction between hNHE1cdt and iaERK2 uncovered here extends this concept. For hNHE1, the multi-site interaction may function in a manner analogous, but not identical, to a fuzzy complex [59, 60], where several binding sites are at play concomitantly, yet with none of the interactions seemingly affecting each other. The “hot potato hypothesis” was originally suggested by Perham in 1975 to describe the handover of substrates and intermediates in multi-enzyme complexes [61]. Indeed, the non-cooperative, multi-site scaffold interaction between hNHE1 and ERK2 may function similarly to holding a hot potato; the sites do not cooperate to increase the affinity, but will “shuffle” dynamically with sites being sometimes off, sometimes on, and hence we term this type of scaffold interaction a “shuffle complex” (Fig. 7).

NHE1 is, to the best of our knowledge, the first membrane protein described to scaffold members of the MAPK pathway, spanning all four levels of the MAPK hierarchy [33]. Many soluble scaffold proteins acting together with, for example, G protein-coupled receptors (GPCRs) and growth factor receptors, have been described, but detailed interaction data have not provided insight into how scaffolding and regulation are coupled. In the cell, MEKs, phosphatases, and substrates all compete for the D-domain recognition site on ERK2 [62], and it is currently not known how NHE1 interacts with the other MAPK members, including MEK, and assembles a signalling complex. As NHE1 acts as a dimer *in vivo* [36, 63], we propose that upon release of the D3-domain from one NHE1 monomer due to competition with MEK, the shuffle complex organization keeps ERK2 in place via scaffolding by D-domains of the other NHE1 subunit in the NHE1 dimer, or by the F-sites (Fig. 7a, b). Furthermore, in a potential cellular complex, the remaining D-domains as well as F-sites will be available for further scaffolding of other members of the MAPK hierarchy (Fig. 7c), as suggested by yeast two-hybrid screens, potentially MEK, although this remains to be explored.

NHE1 is the first example of an ID substrate of ERK2 for which detailed interaction data now exist, and to the best of our knowledge, no other IDP or IDR has to date been experimentally linked to ERK2 phosphorylation or scaffolding. It has been noted that the D-domain of the tyrosine-phosphatase PTP-SL resides in a region with high disorder propensity [64]. Disorder predictions of the nuclear pore protein Tpr (Additional file 7: Figure S7) show its ERK2-interacting F-site to reside in an IDR, similar to the nucleoporin FG-repeat regions and to hNHE1cdt. Thus, it appears that the F-site recruitment site interaction may be frequently exploited by IDPs. However, it remains to be seen whether the multi-site shuffle interaction is a novel canonical IDP/IDR-ERK2 interaction mode or if it is a unique scaffolding function specific to NHE1.

All six putative ERK2 phosphorylation sites of hNHE1cdt were phosphorylated by aERK2 *in vitro* (S693, S723, S726, S771, T779, and S785). Previous *in vivo* phosphoproteomics have mapped phosphorylation at five of these sites, yet without identification of the responsible kinases, and with no information on the sequence of individual phosphorylation events [48, 65]. Further, some of these sites, i.e. S693, T779, and S785, were previously identified upon *in vitro* phosphorylation of NHE1 by ERK2 [29]. No non-canonical phosphorylation was detected in the present study, in contrast with previous reports identifying S766, S770, and S771 as ERK-dependent NHE1 phosphorylation sites [25, 29]. While it is possible that additional complexity may be introduced in the *in vivo* setting, our data underscore the major advantage of NMR for direct identification of phosphorylation sites.

Physiological roles have been proposed for all six phosphorylations, although their interplay and dynamics have never previously been assessed. S723 and S726 (corresponding to S722 and S725 in rabbit NHE1) were reported to be phosphorylated by p38 MAPK in murine pro-B-cells [66], and phosphorylation of S726 was suggested to mediate apoptosis-induced alkalinization by NHE1 [67]. Based on studies of NHE1 mutants expressed in NHE1-deficient CHO cells, S771 was reported to mediate ERK-dependent NHE1 activation [25], and later, S771, T779, and S785, but not S693, S723, or S726, which were assigned roles in ERK-dependent NHE1 phosphorylation after sustained acidosis in renal cells [68]. In *Amphiuma* erythrocytes, phosphorylation of residues corresponding to S693 and S785 (S701 and S783) were detected by MS, where S785 (S783) was constitutively phosphorylated [69]. The precise downstream effects of these phosphorylations are not currently known, but they are likely to both impact NHE1 structural dynamics and hence activity, and to contribute to the scaffolding role of NHE1 in regulation of ERK, hence fine-tuning cellular ERK signaling. Timing of signalling events is crucial for many cellular functions, and phosphorylation events that are interdependent or distributive with very different rate constants are possible ways of controlling signal duration and strength. Indeed, it has been suggested that such temporally ordered phosphorylations serve as platforms for signal integration [55]. Our findings provide evidence for a distinct temporal order of ERK2 phosphorylation of hNHE1cdt with the occurrence of specific intermediates. These intermediates could function as tightly regulated docking sites or thresholds that convert graded signals to switch-like responses [70]. Such dynamics in ERK2 signalling have been observed to affect the half-life of an ERK2 downstream effector, the transcription factor c-Fos [15]. Timed phosphorylation events in hNHE1cdt may therefore similarly partake in control of the ERK2 signal duration. The hNHE1cdt has other confirmed phosphorylation sites than those demonstrated in the present study, and many more putative ones [36], several of which are close to the ERK2 interaction sites, for example S703, phosphorylated by RSK [27]. These sites may mediate interactions with other binding partners, introducing additional layers of complexities, for example of pathway crosstalk.

The close proximity of the primary S693 and T779 phosphorylation sites to the D3-domain and F1-site, respectively, suggests regulatory role(s) for the interaction of hNHE1cdt with, and phosphorylation by, ERK2. Analogously, the first phosphorylation events in hNHE1cdt (S693 and T779) may change the binding mode and/or dynamics with ERK2 in a way that regulates the phosphorylation of succeeding sites, although we showed that they do not act as priming sites. The effects of hNHE1 variants on the phosphorylation kinetics support the hypothesis that all sites are at play within the shuffle complex, where the sites closest to the phosphorylation site are exploited for interaction with ERK2. The current data does not allow us to evaluate whether all six sites are phosphorylated during NHE1 activation. Some sites, and most likely the slowest ones observed here, may only react under certain physiological conditions. We hypothesize that such conditional phosphorylations could be important for a rheostatic regulation of both ERK2 and NHE1. Consequently, the results of this work open a series of new questions, both regarding the generality of shuffle complexes in scaffolding by IDPs, but also concerning the functional roles and spatial and temporal interconnectivity of the six identified phosphorylation sites in hNHE1.

## Conclusions

In this work we have demonstrated that the intrinsically disordered region of hNHE1 acts as a membrane scaffold engaging ERK2 in a multi-site shuffle complex. We show that the interaction is recapitulated *in vivo*, and that co-regulation of hNHE1 and ERK2 manifests in distinct *in vivo* effects on ERK1/2 activity and *in vitro* effects on hNHE1 phosphorylation. Our work provides a molecular mechanism for the important scaffolding function of NHE1, and characterizes a direct interaction between the intrinsically disordered hNHE1cdt and ERK2, leading to hNHE1cdt phosphorylation and regulation of ERK1/2 activity.

## Methods

### Cloning and mutagenesis

The human NHE1 C-terminal distal tail was extended by six residues to M + I680-Q815 (hNHE1cdt; primer sequences presented in Additional file 8). The variants D1D2-(AXA)_2_, D3-AXA, D1D2D3-(AXA)_3_, F1-A, and F2-AA; S693A and T779A were prepared from the WT hNHE1cdt plasmid and the WT full-length hNHE1 in a pcDNA3.1 plasmid using a QuikChange II Kit (Stratagene). Final constructs were confirmed by sequencing (Eurofins MWG Operon).

### Protein expression and purification

The expression and purification of unlabeled human ERK2, and of hNHE1cdt and ^15^N- and ^13^C,^15^N- labeled hNHE1cdt were performed essentially as in [71]. All protein preparations were > 95 % pure judged from SDS-PAGE (Additional file 9: Figure S8). Details are presented in Additional file 8: Supplementary materials and methods.

### Bioinformatics

Putative D-domains and consensus ERK2 phosphorylation sites in the hNHE1cdt were predicted by Scansite 3 [72] and aligned with Clustal Omega [73]. Intrinsic disorder was analysed using PONDR-FIT [74] and DISOPRED 3.1 [75].

### NMR spectroscopy

All NMR spectra were recorded on Varian INOVA 750 MHz or 800 MHz ^1^H NMR spectrometers with a 5 mm triple resonance probe and a Z field gradient at 5 °C. Chemical shift referencing was relative to 4,4-dimethyl-4-silapentane-1-sulfonic acid (DSS), and spectra zero-filled, apodized, Fourier transformed, and baseline-corrected in NMRDraw [76], and analysed manually in CCPN Analysis [77]. Backbone resonance assignments of hNHE1cdt and variants were done at 5 °C using 1.0–1.5 mM samples of ^15^N,^13^C-hNHE1cdt in PBS pH 7.2, 0.5 mM DSS, 10 mM dithiothreitol (DTT), and 10 % (v/v) 99.96 % D_2_O by standard 3D triple resonance experiments as described [35]. Intrinsic random coil referencing was done from assignments of hNHE1cdt in 8 M urea from similar experiments. Chemical shift perturbations of hNHE1cdt WT and variants from interaction with unlabeled iaERK2 were determined using 0.1 mM ^15^N-labeled hNHE1cdt WT or variants in the presence/absence of equimolar iaERK2 and ^15^N,^1^H-HSQC spectral analyses, dialyzed against PBS pH 7.4, 5 mM EDTA, and added 0.5 mM DSS, 2 mM DTT, 10 % (v/v) 99.96 % D_2_O prior to recording. Chemical shift perturbations of hNHE1cdt WT by variation of pH were determined using 0.1 mM ^15^N-labeled hNHE1cdt WT in PBS, 2 mM DTT, 0.5 mM DSS, 10 % (v/v) 99.96 % D_2_O at pH 7.2 and 7.4 and ^15^N,^1^H-HSQC spectral analyses. ^15^N transverse relaxation times (*R*_*2*_) were determined using standard ^15^N,^1^H-HSQCs at a 750 MHz proton frequency field at 5 °C with relaxation decays extracted from a nine-step relaxation delay (0.01, 0.05, 0.09, 0.13, 0.17, 0.19, 0.21, 0.23, and 0.25 s). *R*_*2*_ values were calculated by fitting the height of each peak to a single exponential decay function, and each fit was manually reviewed.

### Native mass spectrometry

Protein samples were dialyzed against 200 mM ammonium acetate supplemented with 0.5 mM DTT. To detect the complex 12 μM ERK2 (MW 42343.7 Da) was mixed with a 4 × molar excess of NHE1cdt (48 μM, 14755.3 Da). Intact mass spectrometry experiments were performed on a Waters SYNAPT HDMS modified for high mass transmission as previously described [78]. Gold-coated capillaries prepared in-house [79] were filled with sample and held at 1.6 kV against a sample cone of 200 V. Trap and transfer collision cells were maintained at 10 V, with an argon collision gas at 6.6 × 10^-2^ mbar. Backing pressure in the early ion optics was increased to 5.5 mbar to improve transmission of high m/z ions. Spectra were assigned using the UniDec software as previously described [46].

### Model of hNHE1-ERK2 complex

The structure of iaERK2 in complex with PEA [PDB:4IZ5] [18] was used for F-site recruitment site modelling and the structure of iaERK2 in complex with a MAP kinase interacting kinase peptide [PDB:4H3Q] [80] for the D-recruitment site modelling. From the templates, the ERK2-NHE1 complex was modelled using Modeler version 9.11 [81], generating an ensemble of 1,000 models. These were clustered using the Linkage algorithm and the average structure from the most populated cluster selected as a final model. In the final model, the remaining linker regions were analysed to assess whether they were able to provide a structure of the complex compatible with the hydrodynamic radius observed experimentally. Since linker locations in the complex were ambiguous, they are not shown in the figure.

### *In vitro* ERK2 phosphorylation assays by NMR

Time course experiments were run at 25 °C. Assignments were transferred to 25 °C by recording ^15^N,^1^H-HSQCs at 5, 10, 15, 20, and 25 °C. NMR samples of 400 μL of 100 μM or 200 μM ^15^N-labeled hNHE1cdt or variants were prepared in PBS buffer, 5 mM EDTA, 5 mM ATP, 15 mM MgCl_2_, 0.01 % (w/v) NaN_3_, 1 mM PMSF, 0.5 mM DSS, 2 mM DTT, 10 % (v/v) 99.96 % D_2_O, pH 7.0. A reference ^15^N,^1^H-HSQC spectrum was recorded before addition of kinase. Phosphorylation was started by addition of 10 μL of 0.1 mg/mL (55 nM, 401,000 units/mg) unlabeled active ERK2 (proteinkinase.de), resulting in a molar excess of hNHE1cdt:ERK2 of 1,800:1. Phosphorylation was monitored from extraction of peak position and volumes from a series of ^15^N,^1^H-HSQCs. Peak intensities were normalized to unperturbed residues (Q815). Kinetics of S693, S771, T779, and S785 phosphorylation were extracted from non-linear least squares fittings of disappearing peaks of the unphosphorylated state and/or appearing peaks of the phosphorylated state, as well as reporting neighbours to single exponentials (S693 (D692, S693, and R700), S771 (S770, S771, and G773), T779 (V777 and T779), and S785 (S785, S787, and S788)). Kinetics of S723 and S726 require fitting to bi-exponentials due to their crosstalk. For this purpose peak intensities reporting on the disappearing unphosphorylated state, the appearing phosphorylated state, and both intermediates I_1_ and I_2_ of both S723 and S726 were fitted. For the comparison of the NHE1cdt variants, one peak was chosen for each site and each variant, i.e. S693, S723, S771^P^, V777 (reporting on T779), and S785. The disappearing peak of S723 reports on both, S723 and S726 phosphorylation. Fully phosphorylated ^15^N,^13^C-hNHE1cdt was assigned from standard 3D triple resonance NMR experiments as above.

### Mammalian cell culture and transfections

AP-1 cells (a kind gift from Dr. S. Grinstein, University of Toronto, ON, Canada), which are CHO-derived cells with no endogenous NHE activity [41] and no recovery from an acid load in the nominal absence of HCO_3_^-^ [41, 82], were used for all experiments in mammalian cells. AP-1 cells were grown at 37 °C, 5 % CO_2_, 95 % humidity in α-Minimum Essential Medium Eagle (Sigma) with 10 % fetal bovine serum, 1 % L-glutamine, 1 % penicillin/streptomycin (Gibco). Every 3–4 days, cells were passaged by gentle trypsination, and only passages 5–35 were used for experiments. WT and variant hNHE1 were expressed in AP-1 cells as in [82]. Transfectants were selected for resistance to 600 μg/ml G418 (Calbiochem), individual clones picked, and hNHE1 expression verified by immunoblotting and immunofluorescence analysis.

### EGF-mediated stimulation of ERK1/2 activity in AP-1 cells

Untransfected AP-1 cells or AP-1 cells expressing WT or variant hNHE1 were grown to ~ 80 % confluence in 10 cm Petri dishes, and incubated for 15 min in absence or presence of 100 ng/ml recombinant human EGF (Sigma). Cells were subsequently lysed and processed for immunoblotting as described below.

### Immunoblotting

Immunoblotting was carried out essentially as in [26]. Antibody descriptions and experimental details are provided in Additional file 8. For quantifications, blots were scanned, and band intensities quantified using Un-Scan-IT Graph Digitizer software (Silk Scientific). The pERK1/2 and ERK1/2 bands were normalized to those of the loading control (tubulin) from the same gel to eliminate gel-to-gel differences, and subsequently, pERK1/2 was taken relative to total ERK1/2 from the same experiment.

### Immunofluorescence analysis

Immunofluorescence analysis was carried out essentially as in [22]. Antibody descriptions and experimental details are provided in Additional file 8. Line scan quantification of immunofluorescence was performed using Olympus image analysis software, as the average pixel intensity at each wavelength across the line indicated. Co-localization was quantified as the percentage of cells with NHE1-ERK1/2 co-localization in both membranes, based on representative immunofluorescence images. Data are shown as mean percentage with SEM error bars, based on analysis of at least 60 cells in three to five independent replicates per condition.

### Proximity ligation assay

Proximity ligation assay was carried out with the Duolink II Detection Reagents Red kit from Sigma Aldrich. AP-1 WT cells were seeded on coverslips the day before assaying. Cells were washed in ice-cold PBS, fixed in 4 % PFA for 20 min on ice, and washed in Duolink II Buffer A. Quenching was carried out in 0.1 M glycine for 15 min followed by permeabilization in 0.5 % Triton X-100. After permeabilization, cells were washed in Duolink II Buffer A and added O-link blocking solution for 30 min. Incubation with primary antibodies for 60 min in a humidity chamber at 37 °C was followed by 60 min incubation with PLA probes diluted 1:5 in Duolink II Antibody Diluent buffer at 37 °C. Coverslips were washed in Duolink II Buffer A and added ligation solution diluted 1:5 for 30 min at 37 °C, followed by wash in Duolink II Buffer A. Amplification solution diluted 1:5 was carried out for 100 min at 37 °C. After amplification, coverslips were washed in Duolink Buffer A, incubated with phalloidin^488^ for 1 h, and treated with DAPI to stain nuclei. Finally, coverslips were washed in Duolink Buffer A, mounted on object glass with mounting buffer, and sealed with nail polish. Imaging was carried out with an Olympus BX-61 epifluorescence microscope using cellSens Dimensions V1.6 software. Images were taken as z-stacks and z-projection images were created. Further image processing and quantification were carried out in ImageJ.

### Data analysis and statistics

Data from mammalian cell culture on NHE1 function, immunofluorescence, and immunoblotting are shown as individual experiments representative of at least *n* = 3, or as means ± standard error of the mean (SEM) as indicated. ANOVA with Tukey post-test, or Student’s *t*-test, as appropriate, were used to test for statistically significant differences, with *p* < 0.05 as the significance level.

### Availability of data and materials

Data supporting the results of this article are available in the Additional files 1, 2, 3, 4, 5, 6, 7, and 9, and further details on the materials and methods can be found in Additional file 8. Backbone assignments of the NHE1cdt have been deposited in the BioMagResBank [BMRB:26755].

## Additional files

Additional file 1: Figure S1. pH effect on chemical shifts of the hNHE1cdt and ERK2 autoactivation and dephosphorylation by HePTP. **(a)** Variation of amide chemical shift by a pH difference of 0.2 units for hNHE1cdt. **(b)** Native PAGE of recombinantly expressed ERK2 reveals < 20 % auto-phosphorylation that is removed upon addition of HePTP. (PDF 1208 kb)

Additional file 2: Figure S2. *In vitro* hNHE1cdt-ERK2 interaction. **(a–b)** Combined chemical shift perturbations Δδ(^15^N,^1^H) and peak intensity ratios of WT hNHE1cdt by interaction with ERK2 fully dephosphorylated by HePTP. **(c)** Combined chemical shift perturbations Δδ(^15^N,^1^H) of hNHE1cdt D3-AXA by iaERK2. **(d–e)** Combined chemical shift perturbations Δδ(^15^N,^1^H) and peak intensity ratios of hNHE1cdt D1D2-(AXA)_2_ by iaERK2. **(f–g)** Combined chemical shift perturbations Δδ(^15^N,^1^H) of hNHE1cdt F1-A and F2-AA by iaERK2. **(h)** Internally urea referenced secondary chemical shifts (ΔδC’) of hNHE1cdt WT. (PDF 399 kb)

Additional file 3: Figure S3. Analysis of the hNHE1cdt-iaERK2 complex by size exclusion chromatography and circular dichroism (CD). **(a)** Size exclusion chromatography profiles of the hNHE1cdt WT (*blue*), iaERK2 (*black*), and a mixture of both (*red*). Subtraction of the individual runs from the mixture identifies a broad underlying peak with elution properties of the complex (*grey*). **(b)** Zoom from panel (a). Fractions taken for SDS-PAGE analysis (see panel (c)) are indicated by *grey bars*. **(c)** Coomassie-stained SDS-PAGE of fractions from individual runs, as indicated in panel (b). **(d)** Mean residual ellipticity CD spectra of hNHE1cdt WT, iaERK2, and mixture reveal no significant gain of secondary structure upon complex formation. (PDF 5632 kb)

Additional file 4: Figure S4. Affinity of the hNHE1cdt to iaERK2. **(a)** Peak behaviour for residues of the D3-domain, the F1-site, and the F2-site upon titration with iaERK2. Q815 is shown as a negative example. **(b)** Plotted peak intensity changes (*top*) and chemical shifts (*bottom*) for residues of the D3-domain, the F1-site, and the F2-site upon titration with iaERK2. The D3-domain has the highest apparent affinity, and single residue non-linear least squares fittings reveals a K_d_^app^ of 35 ± 13 μM, 11 ± 2 μM, and 8 ± 1 μM for L684, T685, and V686, respectively. Global fitting results in a K_d_^app^ of 16 ± 2 μM for the hNHE1 D3-domain. The affinities for the F1- and the F2-sites are lower (>50 μM). (PDF 304 kb)

Additional file 5: Figure S5. Effect of mutations on phosphorylation kinetics. **(a)** Position of docking domains in the NHE1cdt relative to phosphorylation sites. **(b)** Effect of primary phosphosite mutations (S693A and T779A) on NHE1cdt phosphorylation kinetics by aERK2. Mutation on either site leads to increased rates at the other site potentially due to the absence of one sixth of high affinity phosphosites (intramolecular competition). Interestingly, the absence of T779 phosphorylation leads to slower rates at S771 and S785, which are close enough to sense the status of T779. **(c)** Effect of D-domain and F-site mutations on NHE1cdt phosphorylation kinetics by aERK2. The order of phosphorylation events is the same for all variants, yet the rates are modulated by the mutations. (PDF 468 kb)

Additional file 6: Figure S6. NHE1 co-localizes with ERK2. **(a)** Immunofluorescence images of AP-1 cells (hNHE1 D1D2-(AXA)_2_ and D1D2D3-(AXA)_3_) treated or not for 15 min with EGF (100 ng/ml). *Merged images* were zoomed to highlight the co-localization of ERK1/2 and NHE1 (*white arrows*). All other variants are shown in Figure 5. Data are representative of three independent biological replicates. **(b)** Representative line scans across membrane areas of images as in (a). (PDF 2105 kb)

Additional file 7: Figure S7. Disorder prediction for Tpr and nuclear pore proteins. **(a–d)** Intrinsic disorder was predicted by PONDR-FIT and DISOPRED 3.1 for (a) Tpr, (b) Nup50, (c) Nup153, and (d) Nup214, and the positions of FXF repeats were indicated by X. Notably, the great majority of FXF repeats localize to predicted disordered regions. (PDF 353 kb)

Additional file 8: Supplementary materials and methods. (DOCX 25 kb)

Additional file 9: Figure S8. Purity of protein preparations. **(a)** Coomassie-stained SDS-PAGE (Bio-Rad) of hNHE1cdt WT, variants, and iaERK2. A low molecular weight marker (LMW) was used as standard (GE Healthcare). **(b)** Phosphorylation of the hNHE1cdt variants by aERK2 leads to an upward shift on SDS-PAGE towards apparent higher molecular weight relative to the unphosphorylated state. (PDF 1194 kb)
