# Supplementary figures and images for: The human Na+/H+ exchanger 1 is a membrane scaffold protein for extracellular signal-regulated kinase 2
Source: BMC Biol. 2016 Apr 15;14:31. doi: 10.1186/s12915-016-0252-7 (PMC4833948; doi:10.1186/s12915-016-0252-7)

a

hNHE1cdt WT (pH 7.4-7.2)

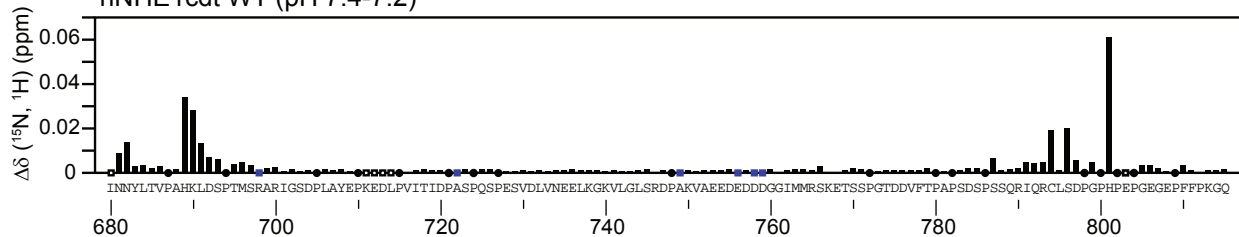

b

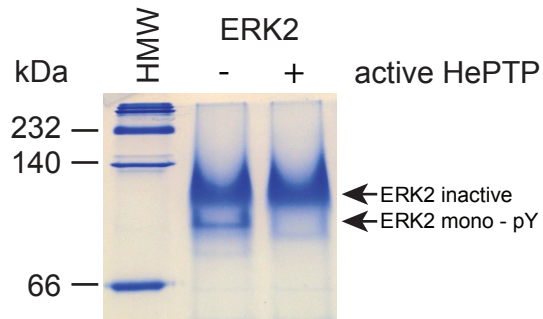

Supplement: Supplementary file 1 — pH effect on chemical shifts of the hNHE1cdt and ERK2 autoactivation and dephosphorylation by HePTP. (a) Variation of amide chemical shift by a pH difference of 0.2 units for hNHE1cdt. (b) Native PAGE of recombinantly expressed ERK2 reveals < 20 % auto-phosphorylation that is removed upon addition of HePTP. (PDF 1208 kb) [file 12915_2016_252_MOESM1_ESM.pdf]

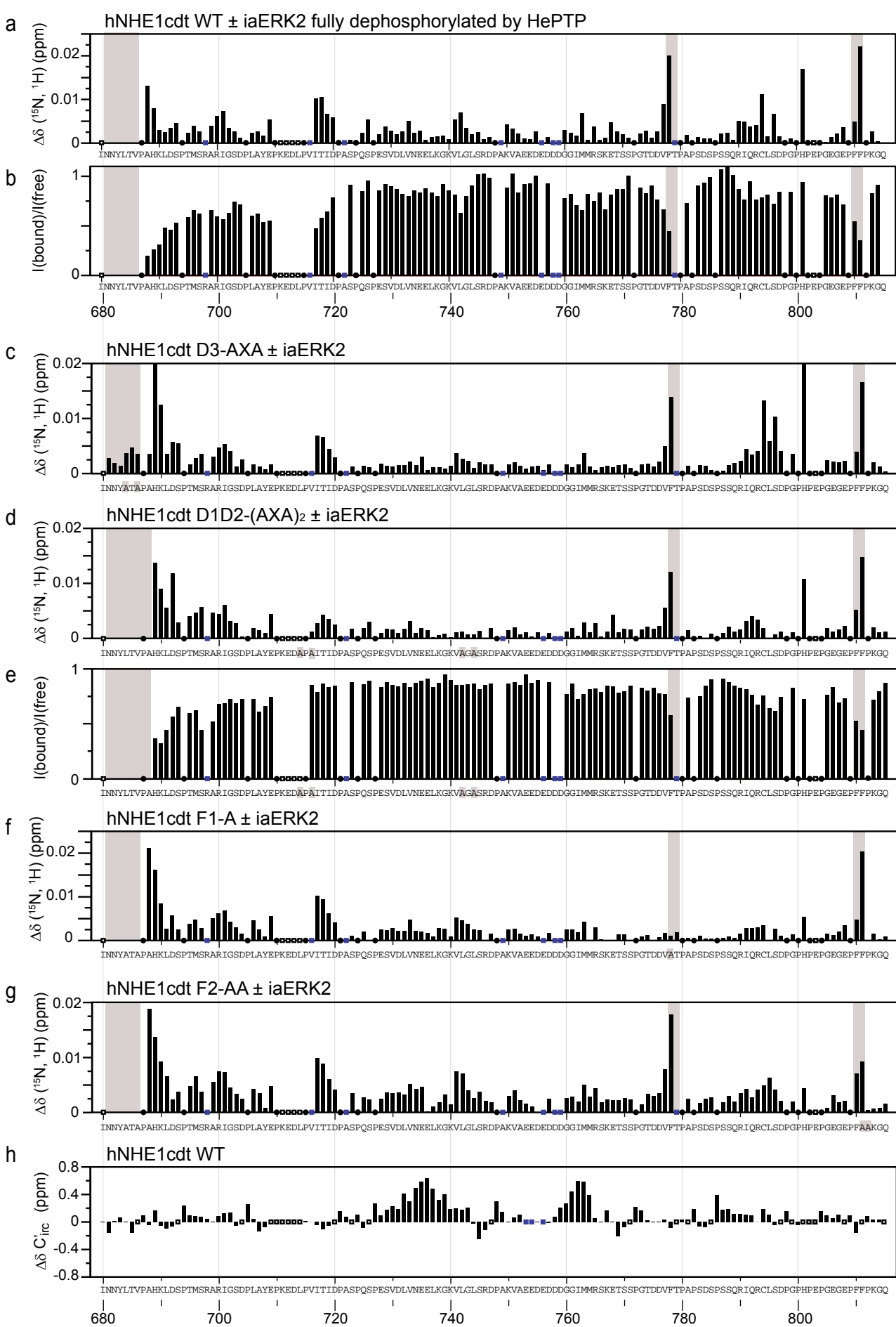

Supplement: Supplementary file 2 — In vitro hNHE1cdt-ERK2 interaction. (a–b) Combined chemical shift perturbations Δδ(15N,1H) and peak intensity ratios of WT hNHE1cdt by interaction with ERK2 fully dephosphorylated by HePTP. (c) Combined chemical shift perturbations Δδ(15N,1H) of hNHE1cdt D3-AXA by iaERK2. (d–e) Combined chemical shift perturbations Δδ(15N,1H) and peak intensity ratios of hNHE1cdt D1D2-(AXA)2 by iaERK2. (f–g) Combined chemical shift perturbations Δδ(15N,1H) of hNHE1cdt F1-A and F2-AA by iaERK2. (h) Internally urea referenced secondary chemical shifts (ΔδC’) of hNHE1cdt WT. (PDF 399 kb) [file 12915_2016_252_MOESM2_ESM.pdf]

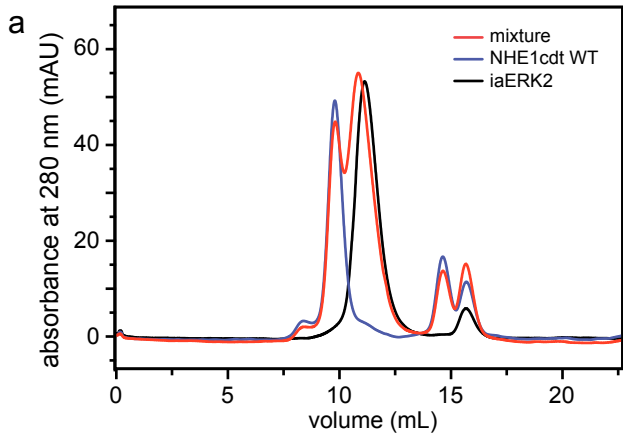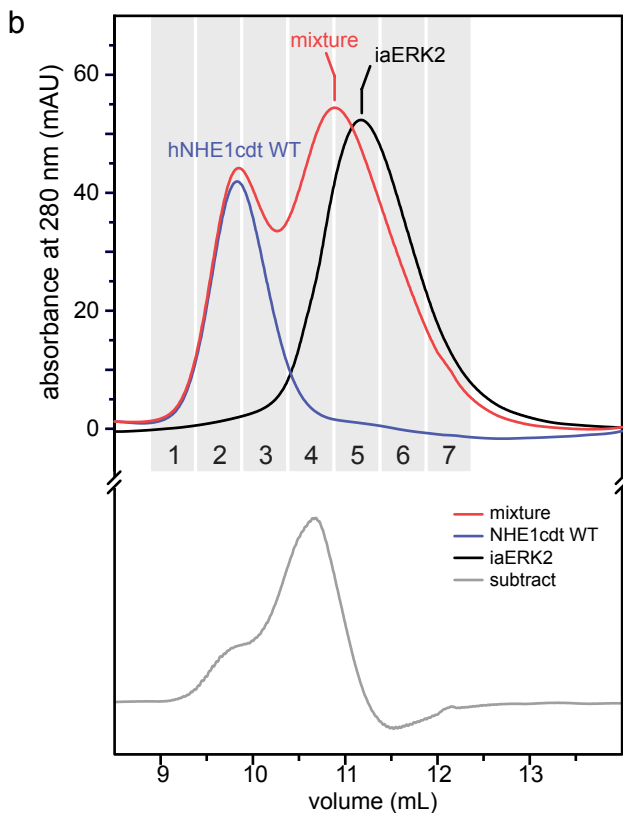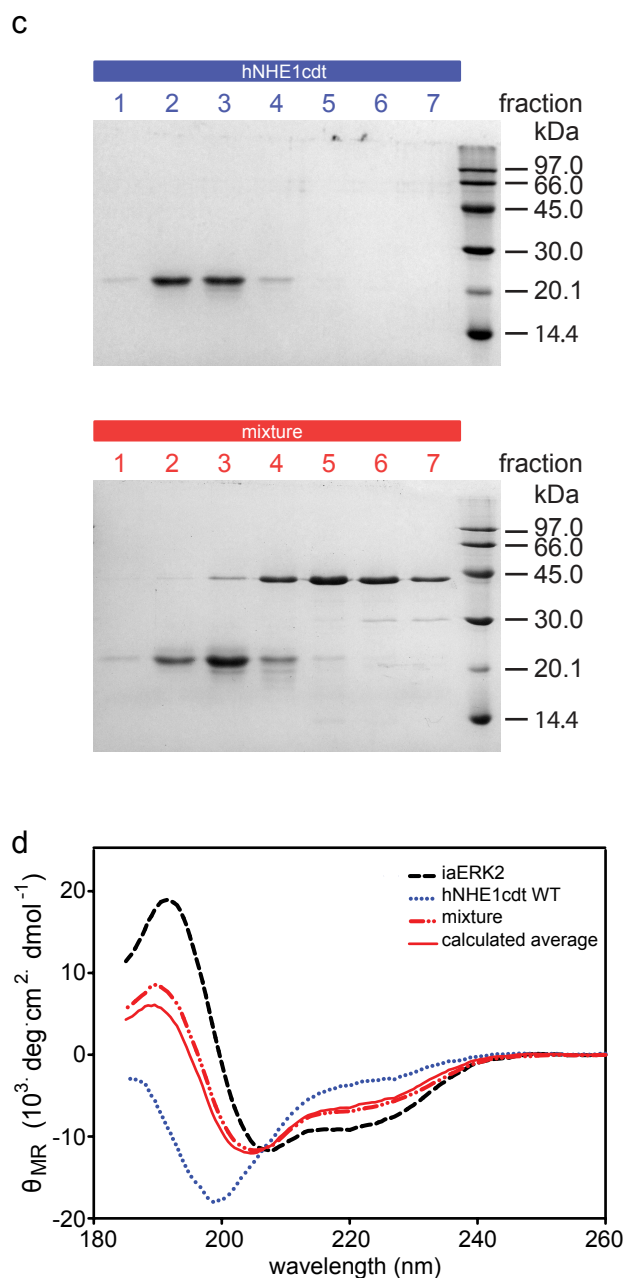

Supplement: Supplementary file 3 — Analysis of the hNHE1cdt-iaERK2 complex by size exclusion chromatography and circular dichroism (CD). (a) Size exclusion chromatography profiles of the hNHE1cdt WT (blue), iaERK2 (black), and a mixture of both (red). Subtraction of the individual runs from the mixture identifies a broad underlying peak with elution properties of the complex (grey). (b) Zoom from panel (a). Fractions taken for SDS-PAGE analysis (see panel (c)) are indicated by grey bars. (c) Coomassie-stained SDS-PAGE of fractions from individual runs, as indicated in panel (b). (d) Mean residual ellipticity CD spectra of hNHE1cdt WT, iaERK2, and mixture reveal no significant gain of secondary structure upon complex formation. (PDF 5632 kb) [file 12915_2016_252_MOESM3_ESM.pdf]

a

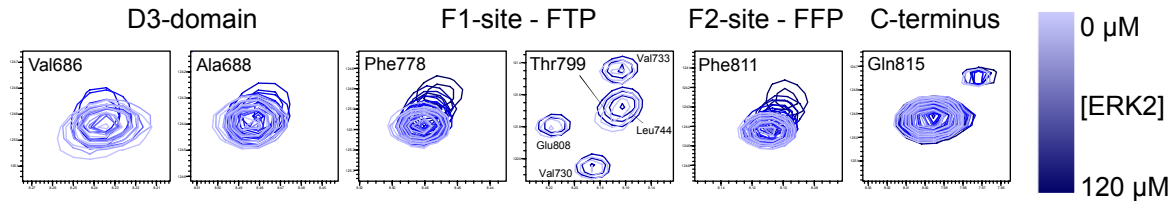

b

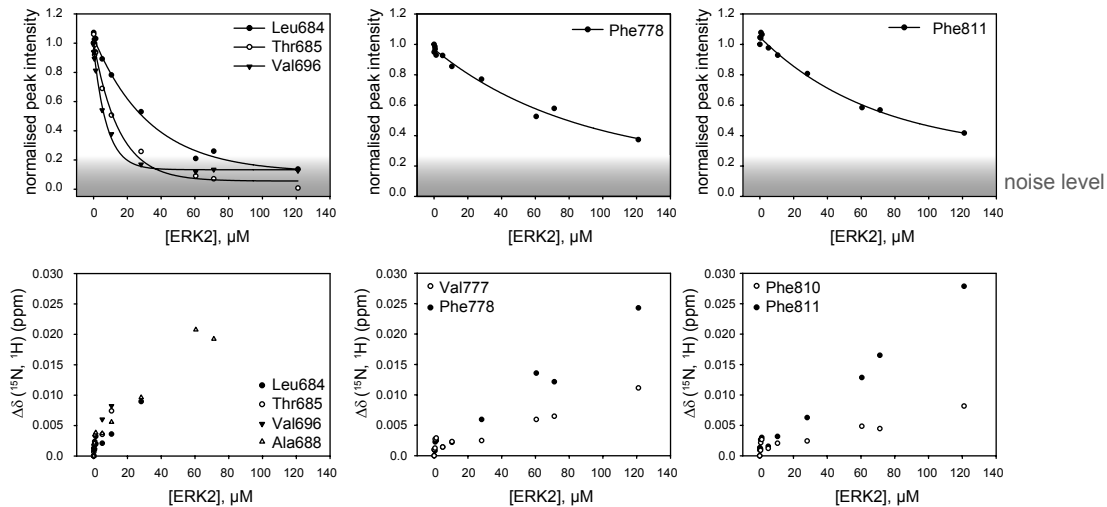

Supplement: Supplementary file 4 — Affinity of the hNHE1cdt to iaERK2. (a) Peak behaviour for residues of the D3-domain, the F1-site, and the F2-site upon titration with iaERK2. Q815 is shown as a negative example. (b) Plotted peak intensity changes (top) and chemical shifts (bottom) for residues of the D3-domain, the F1-site, and the F2-site upon titration with iaERK2. The D3-domain has the highest apparent affinity, and single residue non-linear least squares fittings reveals a Kd app of 35 ± 13 μM, 11 ± 2 μM, and 8 ± 1 μM for L684, T685, and V686, respectively. Global fitting results in a Kd app of 16 ± 2 μM for the hNHE1 D3-domain. The affinities for the F1- and the F2-sites are lower (>50 μM). (PDF 304 kb) [file 12915_2016_252_MOESM4_ESM.pdf]

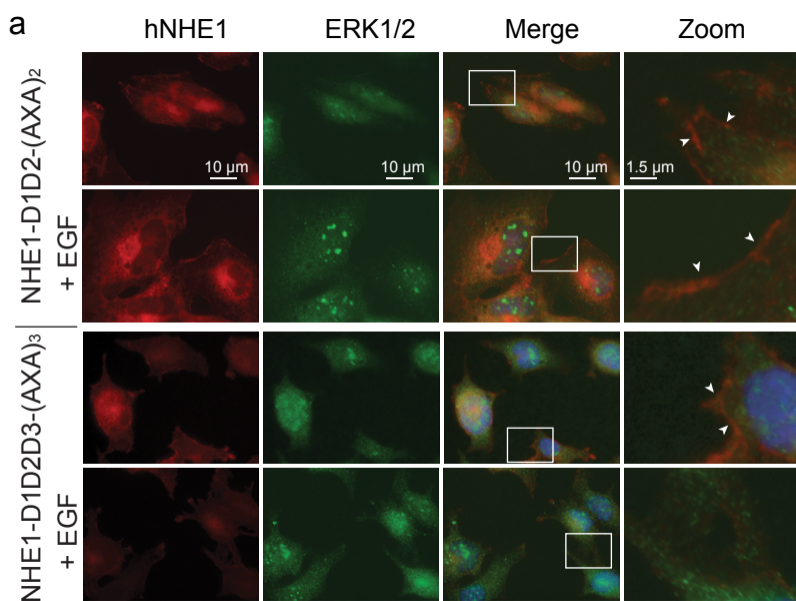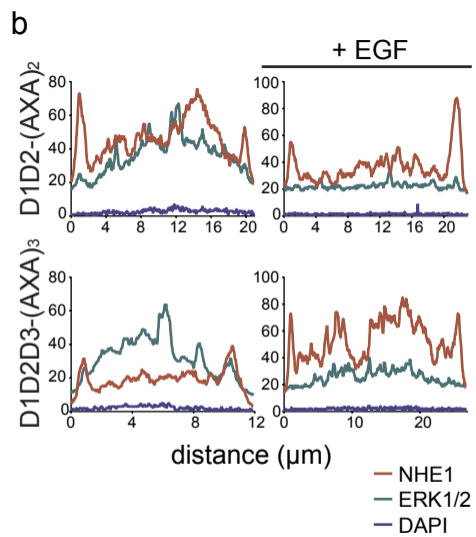

Supplement: Supplementary file 6 — NHE1 co-localizes with ERK2. (a) Immunofluorescence images of AP-1 cells (hNHE1 D1D2-(AXA)2 and D1D2D3-(AXA)3) treated or not for 15 min with EGF (100 ng/ml). Merged images were zoomed to highlight the co-localization of ERK1/2 and NHE1 (white arrows). All other variants are shown in Figure 5. Data are representative of three independent biological replicates. (b) Representative line scans across membrane areas of images as in (a). (PDF 2105 kb) [file 12915_2016_252_MOESM6_ESM.pdf]

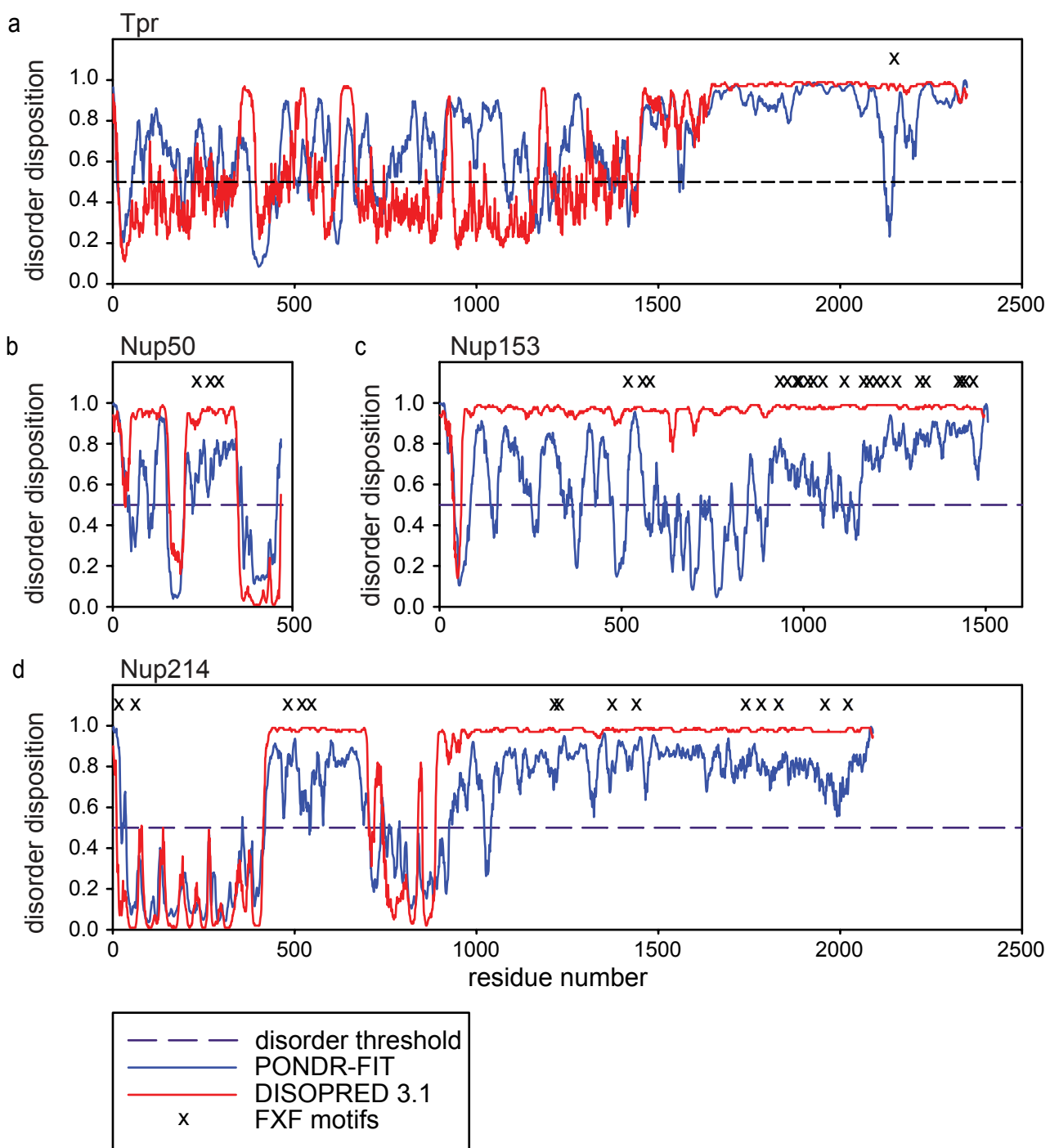

Supplement: Supplementary file 7 — Disorder prediction for Tpr and nuclear pore proteins. (a–d) Intrinsic disorder was predicted by PONDR-FIT and DISOPRED 3.1 for (a) Tpr, (b) Nup50, (c) Nup153, and (d) Nup214, and the positions of FXF repeats were indicated by X. Notably, the great majority of FXF repeats localize to predicted disordered regions. (PDF 353 kb) [file 12915_2016_252_MOESM7_ESM.pdf]

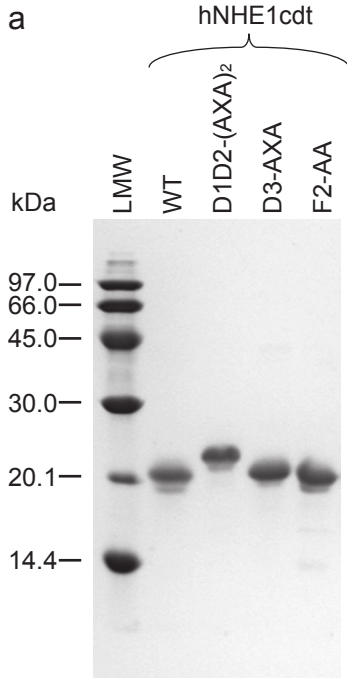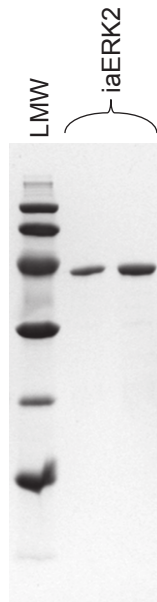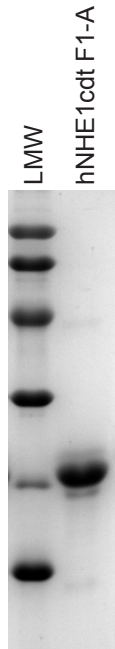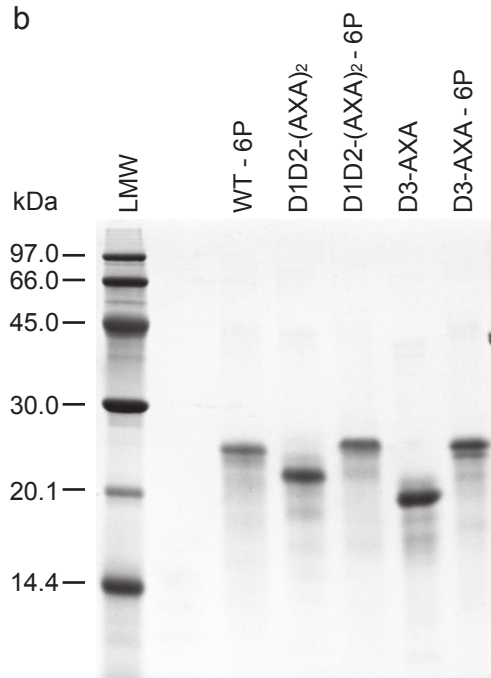

Supplement: Supplementary file 9 — Purity of protein preparations. (a) Coomassie-stained SDS-PAGE (Bio-Rad) of hNHE1cdt WT, variants, and iaERK2. A low molecular weight marker (LMW) was used as standard (GE Healthcare). (b) Phosphorylation of the hNHE1cdt variants by aERK2 leads to an upward shift on SDS-PAGE towards apparent higher molecular weight relative to the unphosphorylated state. (PDF 1194 kb) [file 12915_2016_252_MOESM9_ESM.pdf]
