# Supplementary material for: The human Na+/H+ exchanger 1 is a membrane scaffold protein for extracellular signal-regulated kinase 2
Source: BMC Biol. 2016 Apr 15;14:31. doi: 10.1186/s12915-016-0252-7 (PMC4833948; doi:10.1186/s12915-016-0252-7)
Supplement: Supplementary file 5 — Effect of mutations on phosphorylation kinetics. (a) Position of docking domains in the NHE1cdt relative to phosphorylation sites. (b) Effect of primary phosphosite mutations (S693A and T779A) on NHE1cdt phosphorylation kinetics by aERK2. Mutation on either site leads to increased rates at the other site potentially due to the absence of one sixth of high affinity phosphosites (intramolecular competition). Interestingly, the absence of T779 phosphorylation leads to slower rates at S771 and S785, which are close enough to sense the status of T779. (c) Effect of D-domain and F-site mutations on NHE1cdt phosphorylation kinetics by aERK2. The order of phosphorylation events is the same for all variants, yet the rates are modulated by the mutations. (PDF 468 kb) [file 12915_2016_252_MOESM5_ESM.pdf]

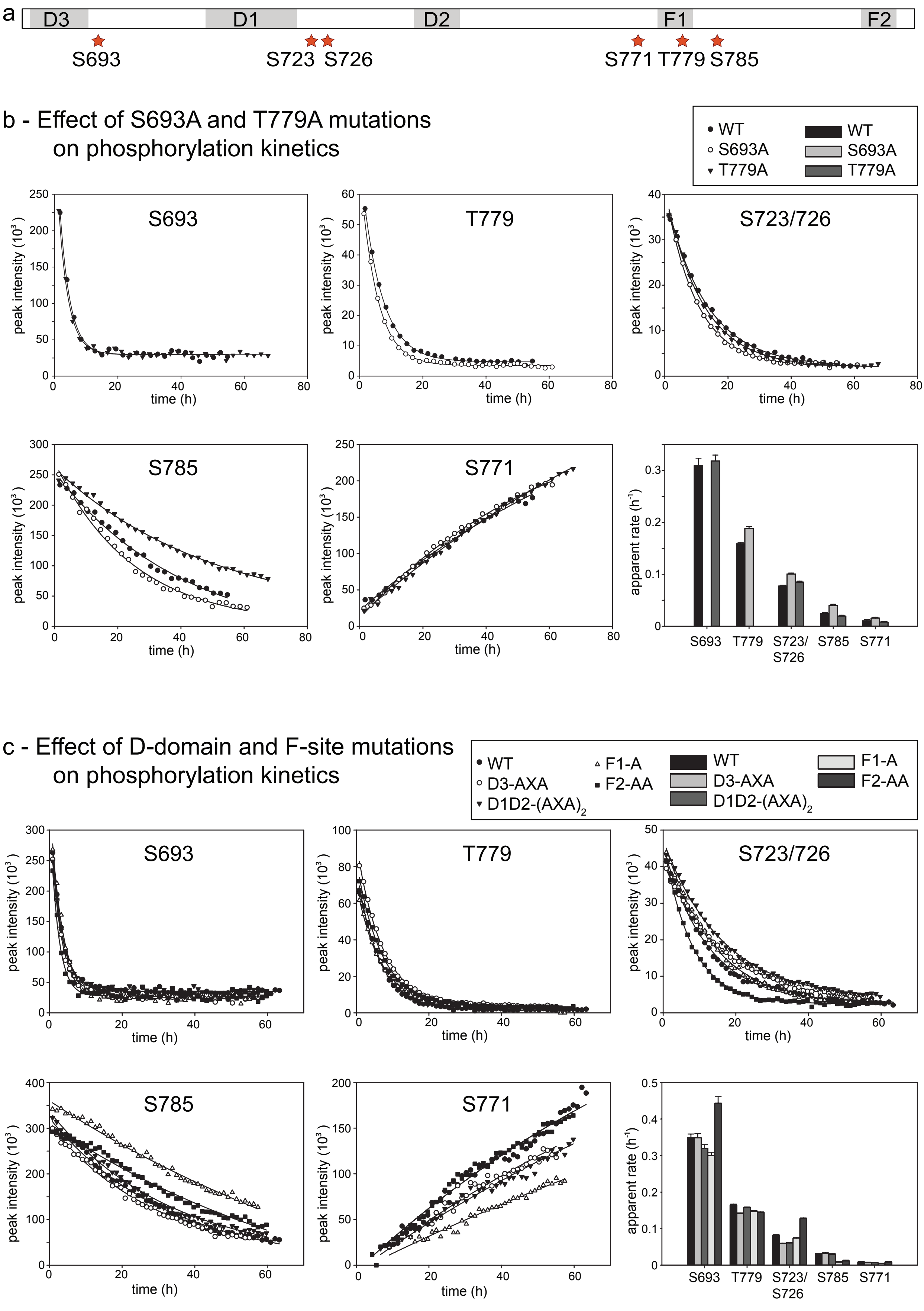
a
D3
D1
D2
F1
F2
S693
S723
S726
S771
T779
S785
b - Effect of S693A and T779A mutations on phosphorylation kinetics
WT
S693A
T779A
S693
T779
S723/726
S785
S771
peak intensity (10^3)
time (h)
apparent rate (h^{-1})
S723/
S726
c - Effect of D-domain and F-site mutations on phosphorylation kinetics
WT
D3-AXA
D1D2-(AXA)$_2$
F1-A
F2-AA
