# Supplementary material for: The human Na+/H+ exchanger 1 is a membrane scaffold protein for extracellular signal-regulated kinase 2
Source: BMC Biol. 2016 Apr 15;14:31. doi: 10.1186/s12915-016-0252-7 (PMC4833948; doi:10.1186/s12915-016-0252-7)
Supplement: Supplementary file 8 — Supplementary materials and methods. (DOCX 25 kb) [file 12915_2016_252_MOESM8_ESM.docx]

**Additional file 8: Supplementary Materials and Methods**

***NHE1 Primers*** - Extension forward primer: 5’-GGAGCATATGATCAACAACTACC-3’ (NdeI), reverse primer: 5’-GTGGCTAGCTCACTGCCCCTTGGGGAAG (NheI) (restriction sites are underlined and denoted in brackets).

Mutagenic primers used are: D1-AXA (_714_LPV-APA): 5’-TATGAGCCGAAGGAGGACGCGCCTGCCA, D2-AXA (_742_LGL-AGA): 5’-GAAGGGCAAAGTCGCAGGGGCGAGLLGG, D3-AXA (_684_LTV-ATA): 5’-GATCAACAACTACGCGACGGCGCCAGCCCACAAG, F1-A (F778A): 5’-CCGACGATGTCGCCACCCCCGCGCCCAG, F2-AA (_811_FP-AA): 5’-GGAGAACCGTTCGCCGCCAAGGGGCAGTG, S693A 5’-CCACAAGCTGGACGCACCCACCATGTC, and T779A 5’-CGATGTCTTCGCCCCCGCGCCCAGTGAC.

***Expression and purification of hNHE1*cdt *WT and variants* -** Expression and purification of unlabeled, ^15^N-labeled and ^13^C, ^15^N-double labelled hNHE1cdt were performed as follows: the supernatant from sonication was heated in an 80°C water bath for 10min under fast stirring, followed by rapid cooling and incubation on ice for 10min. Precipitants were removed by centrifugation (15 000 g, 15min at 4 °C) and DNA contaminations by protamine sulfate precipitation. The supernatant was dialyzed for anion exchange by a HiTrap Q FF column (GE-Healthcare), followed by size exclusion chromatography on a Superdex 75 10/300 GL (GE healthcare). The purity of all protein preparations was >95% as judged from SDS-PAGE (Additional file 8: Figure S8).

***Expression and purification of human inactive ERK2* –** Expression of recombinant inactive ERK2 (iaERK2) from the expression vector pJexpress414 encoding *E. coli* codon optimized human H_6_-tagged ERK2 (DNA 2.0) was done in *E. coli* BL21 (DE3) cells in 1 L Luria Bertani medium grown at 37 °C, 180 rpm until OD_600_= ~0.6. Protein expression was induced using 0.4 mM isopropyl b-d-1-thiogalactopyranoside (IPTG) for 4h. Cells were harvested by centrifugation at 5 000 g and broken by sonication in 50 mL ice-cold 50 mM sodium phosphate buffer pH 8.0, 0.3 M NaCl, 5 mM imidazole, including one EDTA-free protease inhibitor tablet (Roche). The supernatant was directly applied to a Ni-NTA column (GE Healthcare), washed with 5 column volumes (CV) of 50 mM Na_2_HPO_4_ pH 8.0, 0.3 M NaCl, 5 mM imidazole and iaERK2 eluted with 5 CV of 50 mM Na_2_HPO_4_ pH 8.0, 0.3 M NaCl, 500 mM imidazole. Pooled fractions were directly applied to a size exclusion column, HiLoad Superdex 75 26/60 (GE Healthcare), equilibrated in PBS, 5 mM EDTA, pH 7.4. Purity of iaERK2 batches were >95% as judged by SDS-PAGE (Additional file 8: Figure S8). Auto-phosphorylation of ERK2 was estimated by native PAGE (15% Tris glycine). ERK2 was de-phosphorylated by addition of 1:2000 HePTP (Nordic Biosite) in PBS, 5 mM EDTA, 2 mM DTT, 15 mM MgCl_2_ and incubation at 30°C for 1h. HePTP was removed by glutathione resin.

***Determination of K_d_^app^ by NMR*** **-** Titrations were performed by keeping the concentration of WT hNHE1cdt constant (50 µM) and increasing iaERK2 from 0-120 µM. Sample conditions were PBS pH 7.4, 5 mM EDTA, 10 mM DTT, 0.5 mM DSS, and 10% (v/v) 99.96% D_2_O. ^15^N,^1^H-HSQC spectra were recorded for each step at 5°C. For determination of *K*_d_^app^’s the peak volumes from residues in the D3-domain were normalized and plotted against increasing ERK2 concentration and single residue non-linear least squares fits performed using the residues L684, T685, and V686, followed by a global fit of all three residues. All data fittings were done in SigmaPlot.

***Size exclusion chromatography (SEC)*** – Superdex 75 16/85 was equilibrated with two column volumes (CV) of PBS, 5 mM EDTA, 1 mM β-mercapto-ethanol, pH 7.4. hNHE1cdt WT and iaERK2 were dialyzed against running buffer prior to loading. Samples loaded were 500 µL of 20 µM iaERK2, 500 µL 300 µM hNHE1cdt WT, and a mixture of both (500 µL of 20 µM iaERK2 and 300 µM hNHE1cdt WT, pre-incubated for 2h). The column was run with 0.5 mL/min for 1.5 CV at room temperature.

***Circular Dichroism Spectroscopy*** - Far-UV CD measurements were recorded on a Jasco J-810 spectropolarimeter with Peltier control at 10°C using a 0.1 mm path length, from 250 to 195 nm with a scan speed of 10 nm/min, 10 accumulations, and with a data pitch of 0.5 nm. Protein concentrations were 100 µM iaERK2, 200 µM hNHE1cdt WT, and a mixture of both. Sample conditions were PBS, 5 mM EDTA, 1 mM DTT, pH 7.2. Spectra were buffer corrected and converted to mean residual ellipticity.

***Immunoblotting*** *-* Briefly, cells were grown to ~80% confluence in 10 cm Petri dishes, washed in ice-cold PBS, lysed in boiling lysis buffer (1% SDS, 10 mM Tris HCl, pH 7.5), sonicated, and centrifuged to clear debris. Identical amounts of protein (15-25 μg/well) diluted in NuPAGE LDS sample buffer (Lifetech technologies) were boiled for 5 min, separated on NuPage 10% bis-tris gels, and transferred to nitrocellulose membranes using the Novex gel system (Novex, San Diego, CA). Membranes were stained with Ponceau S to confirm equal loading, blocked for 1 h at 37ºC in 120 mM NaCl, 10 mM Tris HCl, 5% nonfat dry milk, and incubated with the relevant primary antibodies in blocking buffer overnight at 4°C. After washing in TBS + 0.1% Tween-20, membranes were incubated with alkaline phosphatase-conjugated secondary antibodies (1:5000, Sigma), washed, and visualized using BCIP/NBT. Densitometric analysis was performed using UN-SCAN-IT software.

***Antibodies for PLA, Immunoblotting and Immunofluorescence analysis***

Rabbit polyclonal ERK1/2 and phospho-T202/Y204 (ERK1)/T185/Y187 (ERK2) antibodies were from Cell Signaling Technology #9102 and #9101 (RRID: AB_330744 and AB_2315036) respectively. Both polyclonal antibodies were produced by immunizing animals with a synthetic peptide derived from a sequence in the C-terminus of rat p44 MAP Kinase (in the ERK1/2 case) and a phospho-peptide corresponding to residues surrounding Thr202/Tyr204 of human p44 MAP kinase. The dilution used for immunoblotting experiments was 1:1000 for both antibodies and the validation was carried out by obtaining only two bands in the blots. Mouse monoclonal anti-beta-actin antibody was from Sigma (A5441, RRID: AB_476744). This antibody was derived from the AC-15 hybridoma produced by the fusion of mouse myeloma cells and splenocytes from an immunized mouse. The dilution used for immunoblotting experiments was 1:10.000 and only one band was observed in the blots.

Rabbit polyclonal NHE1 antibody use for immunofluorescence analysis was a kind gift from M. Musch (University of Chicago, USA). Affinity-purified rabbit polyclonal antibody XB-17 raised against amino acids 639–746 of the cytoplasmic region of human NHE1. The dilution used for immunofluorescence experiments was 1:100 and the validation was by comparing to a nonspecific negative control antibody. Mouse ERK1/2 monoclonal antibody was from Cell signaling Technology (#4696, RRID: AB_390780), produced by immunizing animals with a synthetic peptide corresponding to the sequence of p42 MAP kinase. The dilution used for immunofluorescence experiments was 1:100 and the validation was by comparing to a nonspecific negative control antibody .

***Immunofluorescence analysis of hNHE1 and ERK*** *–* The AP1 cells were a kind gift from S. Grinstein, Hospital for Sick Children, Toronto, Canada. Cells used in these experiments are Chinese Hamster Ovary (CHO)-derived cells without - endogenous NHE1 activity and generated used a proton + suicide technique, where the CHO cells were mutated with methane sulfonate and loaded with Li ^+^. During the experiments, cells were tested for mycoplasma contamination every 3 months. For immunofluorescence experiments, AP1 cells were grown on 12 mm round glass coverslips were fixed in 2% paraformaldehyde (15 min room temperature, 30 min on ice), washed in TBS, permeabilized for 5 min (0.5% Triton X-100 in TBS), blocked for 30 min (5% BSA in TBS + 0.1% Tween20=TBST), incubated with primary antibodies against NHE1 and ERK1/2 (antibody details see above) in TBST + 1% BSA overnight at 4°C, washed in TBST, and with the appropriate AlexaFluor488- or AlexaFluor568 conjugated secondary antibodies (1:600 in TBS + 1 % BSA) for 1 h, followed by washing in TBST, and mounting in N-propyl-galleate mounting medium (2% w/v in PBS/glycerol). DAPI was added for 3 min following incubation with the secondary antibody to stain nuclei. Cells were visualized using the 60X/1.35 NA objective of an Olympus Bx63 epifluorescence microscope. No or negligible labelling was seen in the absence of primary antibody or in untransfected AP-1 cells. Overlays and brightness/contrast adjustment was carried out using Adobe Photoshop software. No other image adjustment was performed.
